# pH-Driven β_2_AR Dynamics Reveal Loop-Mediated Allosteric Communication

**DOI:** 10.1021/acsomega.5c12434

**Published:** 2026-02-12

**Authors:** Nuray Sogunmez Erdogan, E. Demet Akten

**Affiliations:** † 52974 Kadir Has University, Faculty of Engineering and Natural Sciences, Molecular Biology and Genetics, Istanbul 34083, Türkiye; ‡ Kadir Has University, Faculty of Engineering and Natural Sciences, Bioinformatics and Genetics, Istanbul 34083, Türkiye

## Abstract

Membrane protein structure and dynamics are highly sensitive to environmental conditions, including changes in pH that can alter the protonation states of ionizable residues and, in turn, influence local electrostatics and stability. Constant-pH molecular dynamics (CpHMD) provides a framework to explore such effects by allowing dynamic proton exchange during simulations. Here, we applied CpHMD at pH:6.5, 7.0, and 8.0, alongside conventional MD, to examine how pH variations may influence the local conformational behaviors of the β_2_-adrenergic receptor (β_2_AR). During the 1.2-μs-long total simulation, loop regions rich in titratable residues, particularly ICL3 and ECL2, showed the strongest responses to protonation changes. CpHMD trajectories suggested a pH-dependent redistribution of loop flexibility and hydrogen-bonding patterns, producing a “see-saw-like” effect, while fixed-protonation Control runs showed more constrained behavior. Across all simulations, the key GPCR microswitches, such as the ionic lock, the Y–Y gate, the NPxxY and PIF motifs, and the Trp286–Phe290 toggle pair, stayed within the ranges expected for an inactive receptor. This suggests that pH changes mainly influence local loop motions in the inactive receptor without pushing it toward activation-like states. Finally, mutual information analysis on both Cα atoms and dihedral angles revealed altered communication between the extracellular and intracellular loops under different pH environments. While limited in time scale, these results provide a computational perspective on how protonation dynamics can modulate the GPCR behavior and highlight the value of incorporating pH effects in molecular-level investigations.

## Graphical abstract

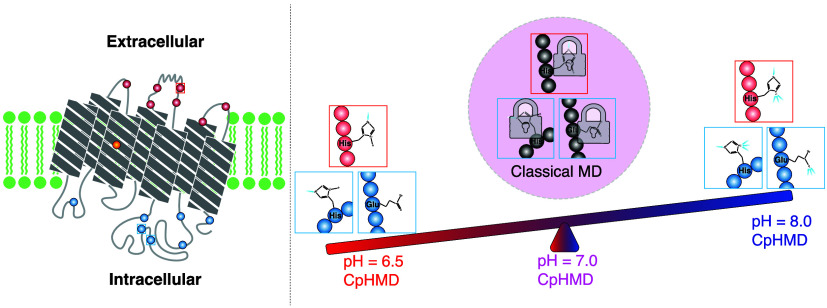

## Introduction

Living organisms must maintain homeostasis, including regulation of pH and temperature for survival.^1^ Although pH homeostasis is vital for physiological functions, pH levels vary across different organs and organelles, such as the Golgi apparatus, mitochondria, and blood plasma,^2^ to ensure optimal enzyme and protein function and to protect against pathogens.^2–6^ Organisms can compensate for minor pH changes. However, extreme conditions or CO_2_ imbalances, electrolytes, or weak acids/bases can disrupt pH regulation, potentially leading to metabolic disorders such as acidosis or alkalosis.^7–11^

pH balance is particularly important for the proper functioning of G-protein-coupled receptors (GPCRs),^12^ which are involved in various physiological processes including smell and vision,^13,14^ behavioral regulation,^15,16^ immune system and inflammation activity,^17^ stimulation of the autonomic nervous system,^18^ and apoptosis.^19^ Therefore, GPCRs are important targets for drug discovery, accounting for almost half of the pharmaceutical market.^20^ As a well-known member of the GPCR family, the β_2_ adrenergic receptor (β_2_AR) is highly expressed in lung and cardiac tissues and is associated with diseases such as asthma, heart failure, and cancer.^21–24^

The structural stability and activation propensity of β_2_AR are modulated by the protonation states of the titratable residues, which makes the receptor sensitive to changes in its microenvironment.^12,25,26^ Additionally, the local membrane charge was shown to bias the β_2_AR coupling (Gs vs Gi_3_).^27^ Therefore, understanding how pH and protonation state alterations modulate conformational changes is critical to comprehend the behavior of β_2_AR under different physiological conditions and to guide the development of targeted therapies that can modulate its activity in varying pH environments.

Respiratory pH levels have been clinically associated with diseases such as chronic obstructive pulmonary disease,^7,28,29^ acute and chronic asthma,^30,31^ and cystic fibrosis.^32^ β_2_AR, predominantly expressed in lung and cardiac tissues, was tagged with the fluorescein biomarker 7-nitro-1,2,3-benzoxadiazole (NBD), revealing its distinct conformational states between pH:6.5 and pH:8.0.^12^ Although the agonist affinity was lower at pH:6.5, the agonist-induced fluorescence change was twice as large as that observed at pH:8.0. This finding was further supported by functional studies, which showed an increase in β_2_AR-mediated *Gα* activation at pH:6.5 compared to pH:8.0, suggesting that pH-dependent protonation promotes higher basal activity by destabilizing the inactive conformation of the receptor.^12^ These experimental findings provide information about the dynamic properties of the receptor and its binding partners under varying pH conditions. However, a deeper understanding of residue-level protonation changes is essential to clarifying the mechanisms underlying these pH-dependent conformational shifts and their coupled interactions.

Although experimental methods have inherent limitations, computational techniques, such as molecular dynamics (MD) simulations, offer valuable insights into protein behavior.^33,34^ High-resolution crystal structures and molecular modeling approaches, together with conventional MD simulations, have significantly advanced our understanding of protein structure, dynamics, and drug interactions.^35–38^ These computational tools can reveal detailed information that often exceeds the experimental capabilities, leading to novel discoveries in protein behavior and drug development.^39^

Among these approaches, conventional MD can effectively capture a wide range of molecular interactions.^34,37^ However, standard MD approaches often fail to accurately model environmental pH or protonation state changes and cannot directly predict microscopic p*K*_*a*_ values of titratable residues, which are essential for understanding pH-dependent macromolecular behavior.^40^ Quantum mechanics-based methods can, in principle, resolve such protonation equilibria, but their computational cost makes them impractical for simulating large-scale conformational changes in membrane proteins.^37^ By contrast, advances in MD, particularly the development of constant-pH molecular dynamics (CpHMD), now enable efficient sampling of pH-dependent protonation changes together with protein conformational dynamics, providing an attractive balance between precision and scalability.^40,41^ This comes at a higher computational cost compared to conventional MD,^42^ but remains far more applicable than quantum-based methods and captures pH-driven conformational dynamics of proteins.

Recent research has employed CpHMD simulations to explore how variations in the protonation state impact protein dynamics. For example, Pieri et al.^43^ combined CpHMD with other computational techniques to pinpoint residues responsible for pH-dependent light absorption in Anabaena Sensory Rhodopsin. Vo et al.^44^ used CpHMD together with weighted-ensemble simulations to investigate the binding behavior of fentanyl to the μ-opioid receptor. Li et al.^45^ applied CpHMD to determine the p*K*_*a*_ values and examined proton-coupled transport mechanisms in a peptide transporter. Jansen et al.^46^ used CpHMD to examine the activation process of the GLIC ion channel, uncovering shifts in the protonation state and pH-driven structural rearrangements. Together, these studies highlight CpHMD’s ability to provide crucial insights into protein responses to pH fluctuations, offering potential solutions to inconsistencies in drug-receptor interaction studies.

Based on recent advances, this study presents a detailed analysis of the inactive state of β_2_AR (Figure 1A) under conventional MD and CpHMD simulations^40^ at three different pH levels (6.5, 7.0, and 8.0) to cover the experimentally relevant window^12^ in which β_2_AR exhibits measurable conformational differences (Figure 1B). Rather than emphasizing large-scale loop motions, we focused on how ligand-binding pocket dynamics, local fluctuations, and conformational diversity respond to changes in protonation equilibria. CpHMD captures these effects by coupling molecular dynamics with Monte Carlo updates of titratable residues’ protonation states (Figure 1C) based on their p*K*_*a*_ values, the ambient pH, and free-energy changes upon protonation or deprotonation.^40^

**Figure 1:**
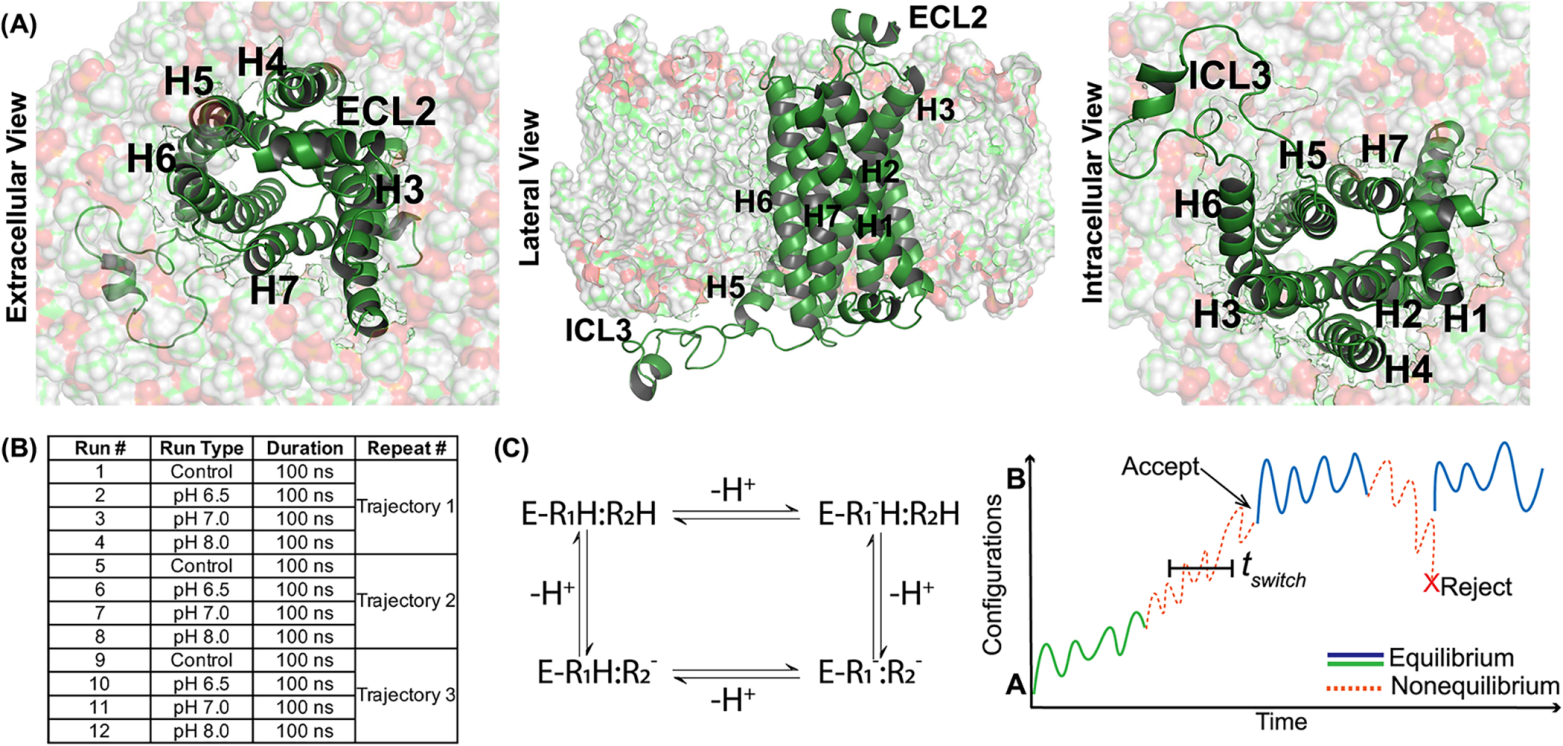
Schematic overview of CpHMD simulations applied to the inactive state of the β_**2**_AR across four different conditions. (A) Structural representations of the β_2_AR are shown from extracellular, lateral, and intracellular perspectives, highlighting the arrangement of helices (H1–H7) and key loops (ECL2, ICL3) within the lipid membrane bilayer. (B) An overview of the study compares conventional MD simulations at neutral pH with CpHMD simulations conducted at pH:6.5, pH:7.0, and pH:8.0, with 3 independent trajectories per condition and a total of 12 runs. Each CpHMD cycle consisted of 100 ps fixed-protonation MD (cpHRun) and 20 ps Monte Carlo protonation attempts (cpHPerSwitch), repeated for 1000 cycles in three replicates at each pH, yielding ∼100 ns trajectories per replica. Control fixed-protonation runs were performed for the same duration to enable direct comparison.^40^ (C) The CpHMD simulation process is depicted, demonstrating how the system alternates between equilibrium and nonequilibrium states to allow protonation and deprotonation events in titratable residues. The Monte Carlo configurational sampling is used to compare the energies of different configurations: if the energy of a protonation switch is lower than the original configuration, the new state is accepted; otherwise, the system retains its original conformation. Adapted from Moe et al.^47^ Copyright 2025 American Chemical Society.

To achieve statistically reliable sampling within feasible resources, we performed three independent 1000-cycle CpHMD runs per condition. Each cycle comprised 100 ps of conventional MD at fixed protonation states to sample conformations, followed by a 20 ps window for Monte Carlo protonation-state change attempts at the target pH, resulting in ∼100 ns per trajectory. Although CpHMD is about 5–10-fold more computationally demanding than conventional MD,^40,42^ this setup provided an effective balance between sampling depth and cost to probe how protonation-state microdynamics respond to modest, physiologically relevant pH shifts.

Simulations revealed that even subtle protonation changes reshape the receptor’s electrostatic landscape, influencing local flexibility and the organization of the binding cavity. Together, these results highlight protonation dynamics as a key yet often overlooked determinant of the β_2_AR structure and energetics, underscoring the limitations of fixed-charge MD in capturing pH-sensitive behavior.

## Methods

### System and Simulation Setup

The system used was the crystal structure of inactive human β_2_AR bound to the partial inverse agonist carazolol (PDB ID: 2RH1).^48^ The crystallization-stabilizing T4-lysozyme and carazolol were removed and replaced with an atomistic model of intracellular loop 3 generated by MODELER,^49^ as described in previous MD studies.^50,51^ The system was embedded in a POPC bilayer, solvated in a box of TIP3P water molecules, and ionized with 42 Na^+^ and 49 Cl^–^ counterions to neutralize the total charge of the system then to adjust the final concentration of 0.15 M.^52^ Long-range electrostatics were treated with PME (grid spacing of 1.0 Å). The periodic box dimensions of 80 Å × 80 Å × 120 Å contained 68,415 atoms. CHARMM36 force field^53^ was used to describe the interaction potential of protein and lipids. Nonbonded interactions used a 12 Å cutoff, a 10 Å switching distance, and a 13.5 Å pairlist distance. Covalent bonds involving hydrogens were constrained with SHAKE, permitting a 2 fs integration time step. Temperature was maintained at 310 K via a Langevin thermostat (damping coefficient 1 ps^–1^), and pressure was regulated at 1 atm using the Langevin piston (piston period 200 fs, piston decay 50 fs) with a flexible cell and constant-area xy-plane with periodic boundary conditions during membrane equilibration and production runs.

System preparation followed a three-step relaxation protocol to avoid artificial membrane distortions. Step 1 involved relaxation of lipid tails, while protein and water atoms were kept fixed according to a fixed-atom mask defined in the PDB β column. This step consisted of 1000 steps of energy minimization followed by 0.5 ns of restrained MD with all nonlipid atoms fixed. Step 2 relaxed both lipids and water while keeping the protein harmonically restrained using force constants (3.0 kcal/mol/Å^2^ scaling). After 1000 steps of minimization and 0.5 ns of MD under the same thermodynamic conditions, the entire system (lipids, water, and protein) was relaxed without any restraints using 1000 steps of minimization and 0.5 ns of unrestrained MD. Following the three-stage relaxation, the system underwent 20 ns of equilibration under NPAT conditions with constant *X*-*Y* area. The lipid bilayer in the system was continuously monitored in the minimization and equilibration steps until it reached the ≈ 63.07 Å^2^ area per lipid ratio, which was in the range of the experimentally reported interval.^54^ To rigorously define the production window, we tracked complementary quality-control metrics over equilibration time, including bilayer thickness (leaflet P–P distance, 36.90 ± 0.30 Å) and bilayer midplane position (midZ, −0.458 ± 0.107 Å).^54^ The protein–bilayer center of mass separation was 6.688 ± 0.098 Å, and the protein–midplane separation was 6.868 ± 0.111 Å, indicating a stable protein–membrane interface throughout the analyzed window.

The production window was defined as the contiguous time interval in which the protein exhibited no systematic drift and maintained stationary variance; only frames within this window were used for all subsequent analyses and statistics (Supporting Figure S2). To further confirm convergence, window-wise distributions of Cα RMSF were calculated in 25 ns intervals across the production phase.^55^ This analysis was performed for both the entire protein and the core region, excluding the ICL3 loop (the longest intracellular loop), to decouple global stability from loop flexibility. Although modest differences existed between independent replicate runs of the same condition, the means of 25 ns long RMSF windows did not show significant changes within the replicates in either global or core-level RMSF distributions, indicating temporal stability of the trajectories (Supporting Figure S2). For each condition (conventional MD and CpHMD at pH:6.5, 7.0, and 8.0), three independent replicates were initiated from the same equilibrated structure but with different velocity seeds at 310 K; CpHMD Monte Carlo titration also used independent random seeds per replicate to ensure genuinely different initial conditions. Unless otherwise stated, summary statistics are reported on replicate-wise block averages within the production window. A total of 12 runs were conducted and analyzed using a time step of 2 fs and a collection interval of 1 ps.

Constant-pH molecular dynamics (CpHMD) simulations were performed by starting from the equilibrated coordinates, velocities, and periodic cell dimensions produced at the end of the equilibration step. Production simulations used the CHARMM36 force field for proteins, lipids, and solvent, supplemented with CpHMD-specific topology and parameter extensions, including constant-pH reference topologies, protonation-state parameter files, and residue-specific configuration libraries (see Data and Software Availability section for details). Trajectories and energies were saved every 10,000 steps (20 ps). All simulations employed a 2 fs integration time step, SHAKE constraints on covalent bonds to hydrogens, and full PME electrostatics with 1.0 Å grid spacing. Short-range nonbonded interactions used a 12 Å cutoff, a 10 Å switching distance, and a 13.5 Å pairlist distance, with van der Waals (vdW) force switching and long-range Lennard–Jones corrections enabled. Temperature was maintained at 310 K using a Langevin thermostat (γ = 1 ps^–1^, applied to heavy atoms), and production trajectories were propagated under constant-area conditions. CpHMD functionality was enabled by sourcing the NAMD CpHMD module and loading the corresponding protonation-state topologies for protein and solvent (Supporting Table S1).

Protonation-state sampling was carried out using the cycle-based NAMD CpHMD protocol.^40,41^ Each cycle consisted of 100 ps of conventional MD propagated at fixed protonation states, followed by a 20 ps Monte Carlo protonation-switching window in which state-change attempts were evaluated for all titratable residues at the target pH. A total of 1000 CpHMD cycles were performed per run, yielding approximately 100 ns of MD sampling per trajectory, and three independent runs were executed for each pH condition (6.5, 7.0, and 8.0). Replica management and synchronization across cycles were enforced using replicaBarrier, ensuring consistent CpHMD progression and pH-dependent protonation dynamics across all trajectories. This protocol enabled fully dynamic sampling of protonation states for all titratable residues throughout the production simulations while maintaining conformational sampling consistent with the designated pH environment. As constant-pH simulations are considerably more computationally demanding than conventional MD, typically requiring multiple additional electrostatic energy evaluations per cycle for each Monte Carlo protonation attempt, leading to an estimated 5–10-fold higher cost,^40,42^ we limited both CpHMD and Control runs to 100 ns for direct comparison.

### Cumulative PCA Calculation

To evaluate the importance of each principal component, the cumulative contribution of variance was calculated as in eq 1

1 $$\mathrm{PC}A_{c}=\frac{\sum_{i=1}^{c}\lambda_{i}}{\sum_{i=1}^{N}\lambda_{i}}$$

where PCA_*c*_ is the cumulative contribution ratio for the top *c* eigenvalues, λ_*i*_ represents the *i*-th eigenvalue, and *N* is the total number of eigenvalues.

### Projection of MD Trajectories onto Principal Components and RMSF Analysis

To evaluate the flexibility of the protein along the dominant mode of motion, RMSF was calculated along PC1. Each trajectory was projected onto PC1 by computing *P* = *X* · *U*_1_, where *X* is the mean-centered and aligned coordinate matrix of C_α_ atoms with dimensions *T* × *N* (*T* frames and *N* atoms), *U*_1_ is the first principal component (PC1) vector of length *N*, and *P* is the resulting *T* × 1 projection of the trajectory onto PC1.

The RMSF was then calculated on the basis of the projected trajectory using eq 2

2 $$\mathrm{RMSF}=\sqrt{\frac{1}{T}\sum_{t=1}^{T}{\left(P_{t}-\left\langle P\right\rangle \right)}^{2}}$$

Here, *P*_*t*_ is the projection of the trajectory onto PC1 in a specific time frame *t* and ⟨*P*⟩ is the average projection value for all time frames.

### Hydrogen Bond Analysis

Hydrogen bonds were detected using a geometric criterion consisting of a donor–hydrogen–acceptor angle ≥ 120° and a hydrogen–acceptor distance ≤ 2.5 Å, considering NH/OH groups as donors and OH/N atoms as acceptors. A given hydrogen bond was included in the analysis only if it occurred in at least 0.1% of the simulation frames (frequency threshold: 0.001) to exclude rare stochastic contacts. For each pH condition, hydrogen bond occurrences were accumulated across the three trajectories and interactions that appeared repeatedly and with high frequency were classified as persistent.

### Average Contact Map

The cutoff distance *R*_*c*_ for heavy atoms (C, N, O, S) was taken as 6.0 Å, below which the atoms were considered to be in contact.^56^ For two residues to be in contact, at least one pair of heavy atoms (one from each residue) must be separated by less than the cutoff distance. The preference for heavy atoms over α carbons was to avoid underestimating side-chain contacts. The formula used for the contact map calculation was the following:

3 $$d_{i,j}=\{\begin{array}{ll} 1, & \mathrm{if}\delta_{i,j}\leq R_{c}.\\ 0, & \mathrm{otherwise}. \end{array}$$

Two residues were considered to be in contact when the distance is less than the threshold in at least 75% of the snapshots.

### Optimizations of the Number of Bins

The probability of observing an atomic fluctuation (*p*(Δ*R*_*i*_(*t*_*k*_))) that occurred at time *t*_*k*_ was calculated as the frequency with which *p*(Δ*R*_*i*_) was observed within the interval *t*_*k*_ ± 0.5*d*, where *d* is the bin size. This bin size was determined by dividing the range Δ*R*_*i*_ (that is, the difference between its minimum and maximum values) by the optimal number of bins. The optimal number of bins for each residue is the one that yields the highest Shannon entropy *H*_*i*_, ensuring the most informative distribution for that residue, defined as:

4 $$H_{i}=-\sum p\left(\Delta R_{i}\left(t_{k}\right)\right)\mathrm{lo}g_{2}\left(p\left(\Delta R_{i}\left(t_{k}\right)\right)\right)$$

where the summation is for *k* different snapshots. The convergence criterion for the number of bins was defined as:

5 $$\frac{H_{i}\left(N_{\mathrm{bins}}+1\right)-H_{i}\left(N_{\mathrm{bins}}\right)}{H_{i}\left(N_{\mathrm{bins}}\right)}\leq 0.02$$

### Mutual Information Analysis

MI, based on atomic fluctuations between residue pairs *i* and *j*, was calculated using the following expression:

6 $$MI_{i,j}=p\left(\Delta R_{i}\left(t_{k}\right),\Delta R_{j}\left(t_{k}\right)\right)\mathrm{lo}g_{2}\frac{p(\Delta R_{i}\left(t_{k}\right),\Delta R_{j}\left(t_{k}\right)}{p\left(\Delta R_{i}\left(t_{k}\right)\right)\cdot p\left(\Delta R_{j}\left(t_{k}\right)\right)}$$

The joint probability *p*(Δ*R*_*i*_(*k*), Δ*R*_*j*_(*l*)) describes the likelihood of simultaneously observing residue *i* in state *k* and residue *j* in state *l*. Mutual information (MI) serves as a non-negative and symmetric measure, indicating the degree of correspondence between the fluctuations of residues *i* and *j*, regardless of their directions. A value of zero for MI suggests that there is no communication or interdependence between the two fluctuations. Cα was selected as a representative atom of each residue.

Similarly, mutual information based on fluctuations in backbone and side torsion angles was expressed as:

7 $$MI_{i,j}=\sum_{\theta_{i}}\sum_{\theta_{j}}p\left(\theta_{i},\theta_{j}\right)\mathrm{lo}g_{2}\frac{p\left(\theta_{i},\theta_{j}\right)}{p\left(\theta_{i}\right)\cdot p\left(\theta_{j}\right)}$$

where *p*(θ_*i*_, θ_*j*_) denotes the joint probability of observing the joint state (θ_*i*_, θ_*j*_) of residues *i* and *j*. Here, θ_*i*_ and θ_*j*_ represent the rotameric states of the backbone ϕ, ψ, and side-chain dihedrals χ_*i*_, where *i* = 1, 2, 3, 4 in residues *i* and *j*, respectively.^57^ Based on the distribution of rotameric states, the number of discrete rotameric states (or bins) for backbone dihedrals was set to 3, whereas for side-chain dihedrals, the number of states varied up to 6 according to the rotamer library.^58^

Mutual information was evaluated at the ensemble level, using the structurally aligned frames from three independently initiated trajectories (7,500 frames per condition), ensuring that the underlying joint probability densities were sampled in a statistically meaningful way.^59,60^

### Finite Sampling Effect

Systematic errors can introduce inaccuracies in the entropy estimation when derived from finite sample sizes. Previous research has demonstrated the necessity of applying corrections to achieve accurate entropy values in such systems.^56,61,62^ This finite sampling error estimation for entropy is given in eq 8

8 $$H^{\mathrm{true}}\approx H^{\mathrm{observed}}+\frac{N_{b}-1}{N_{c}}$$

where *H* ^observed^ is the calculated entropy, *N*_b_ is the number of bins with nonzero probability, and *N*_c_ is the total number of sampled conformations. Since MI_*i*,*j*_ can be written in terms of joint and singlet entropies as in *H*_*i*_ + *H*_*j*_ – *H*_*i*,*j*_, the correction for MI can be derived as in eq 9

9 $$MI^{\mathrm{true}}\approx MI^{\mathrm{observed}}-\frac{N_{b\left(i,j\right)}-N_{b\left(i\right)}-N_{b\left(j\right)}+1}{2N_{c}}$$

where MI^observed^ is the mutual information observed and *N*_b(*i*,*j*)_, *N*_b(*i*)_, and *N*_b(*j*)_ are the corresponding histogram bins to calculate *H*_*i*,*j*_, *H*_*i*_, and *H*_*j*_, respectively.

### Residue Network Analysis

The network of residues was constructed based on mutual information using centrality concepts in graph theory. Let *r*_*i*_(*t*) represent the position of the *C*α atom of residue *i* at time *t*. For a set of “*N*” *C*α atoms, a pairwise distance matrix *D* was calculated for each frame on the trajectory. The distance between residues *i* and *j* was calculated using eq 10

10 $$D_{i,j}=∥r_{i}-r_{j}∥=\sqrt{{\left(x_{i}-x_{j}\right)}^{2}+{\left(y_{i}-y_{j}\right)}^{2}+{\left(z_{i}-z_{j}\right)}^{2}}$$

where *D* is a square and symmetric matrix, therefore *D*_*i*,*j*_ = *D*_*j*,*i*_ and *D*_*i*,*i*_ = 0. An undirected graph *G*(*V*, *E*) was then constructed, where *V* represents the set of nodes (residues) and *E* represents the edges between nodes. An edge (*i*, *j*) was created between the residues *i* and *j* with a sequence distance |*i* – *j*| > 6, if their mutual information (*MI*_*i*,*j*_) exceeds a threshold τ_*mi*_ and their physical distance (*D*_*i*,*j*_) is greater than a specified cutoff τ, as shown in eq 11

11 $$\mathrm{Edg}e_{i,j}=\{\begin{array}{ll} w, & \mathrm{if}\;∥i-j∥>6,D_{i,j}\geq \tau \;\mathrm{and}\;MI_{i,j}\geq \tau_{\mathrm{mi}}\\ 0, & \mathrm{otherwise}. \end{array}$$

The edge weights, *w*, were assigned to the normalized MI values, *MI*_*i*,j_^*^. The degree of centrality for each node (or residue) in the network *i* was calculated by using eq 12

12 $$C_{D\left(i\right)}=\frac{\deg\left(i\right)}{N-1}$$

where deg­(*i*) is the total number of edges connected to node *i*.

Betweenness centrality of node *i* was calculated using eq 13

13 $$C_{B\left(i\right)}=\sum_{s\neq i\neq t}\frac{\sigma_{\mathrm{st}}\left(i\right)}{\sigma_{\mathrm{st}}}$$

where σ_*st*_(*i*) is the number of shortest paths between *s* and *t* passing through *i*, and σ_*st*_ is the total number of shortest paths between *s* and *t*.

## Results and Discussion

### Protonation State Differences Are Directing Protein Dynamics

β_2_AR is a G-protein-coupled receptor that undergoes significant conformational changes that are essential for its role in signal transduction and cellular response.^63,64^ Studying its structural dynamics under varying pH conditions provides insights into how protonation states influence its behavior. While individual trajectories capture unique conformational basins, the ensemble of CpHMD and conventional MD simulations revealed reproducible, pH-dependent variations in local motions and binding pocket dynamics. This comparative framework highlights the collective impact of protonation dynamics on receptor behavior beyond what fixed-charge MD alone can capture.

CpHMD simulations dynamically adjusted the protonation states of ionizable residues in response to the local electrostatic environment, revealing pH-dependent patterns of charge reorganization throughout the receptor. Among the 50 titratable residues evaluated, (Supporting Tables S1–S3), 15 of which exhibited shifts in their protonation fractions during the trajectories (Table 1). Analysis of the running averages of λ-coordinates demonstrated that protonation fractions of these residues reached stable plateaus within the 100 ns CpHMD simulations, indicating that the relevant protonation equilibria were well-sampled under each pH condition (Supporting Figure S1). These residues were primarily located within loop regions and interhelical interfaces, where fluctuating protonation equilibria can influence hydrogen-bond networks, pH-sensitive rearrangements in local flexibility, and long-range information transfer across the receptor.

**Table 1:** Protonation-Deprotonation Fractions of Titratable Residues across Trajectories and pH Conditions^a^

			trajectory 1/2/3		
location	ResID	p*K*_*a*_ (*t* = 0)	pH:6.5	pH:7.0	pH:8.0
Tmemb	122:GLU^(3.41)^	4.4	0/0/0	0/0/0.009	0/0/0
ECL	93:HIS^(2.63)^	6.5	0.971/0.928/0.99	0.856/0.816/0.851	0/0/0.91
	172:HIS^(4.64)^	6.5	1/1/0.566	1/0.744/0.821	0/0.731/0
	178:HIS^(*ECL*2)^	6.5	0.768/0.055/0.875	0.069/0.19/0.146	0.01/0.001/0
	190:CYS^(*ECL*2)^	9.5	1/1/1	1/1/1	1/1/0.998
	192:ASP^(45.51)^	4.0	0.043/0/0	0/0/0	0/0/0
	296:HIS^(6.58)^	6.5	1/0.818/1	1/0.001/0	0/0/0
	305:LYS^(7.31)^	10.4	1/1/1	1/0/1	1/0.986/1
ICL	62:GLU^(*ICL*1)^	4.4	0/0.39/0.383	0/0.171/0	0/0/0
	241:HIS^(*ICL*3)^	6.5	0.97/1/0.932	0.755/0.952/0.963	0.019/0/0
	249:GLU^(*ICL*3)^	4.4	0.998/0/0	0.937/0/0.267	0.002/0/0
	256:HIS^(*ICL*3)^	6.5	0.963/1/1	0.974/0.804/0.863	0/0.823/0.883
	268:GLU^(6.30)^	4.4	0.378/0/0.459	0/0/0	0/0/0
	269:HIS^(6.31)^	6.5	0.004/0.048/0.105	0/0.03/0.003	0/0/0
	338:GLU^(8.56)^	4.4	0.834/0.376/0.972	0/0/0	0/0/0

a Tmemb: transmembrane, ECL: extracellular loop, ICL: intracellular loop.

The analysis of protonation fractions in the CpHMD simulations (Table 1) suggests that the extracellular loop (ECL) regions are the most susceptible to environmental pH changes, largely due to multiple histidine residues located in these domains. Rather than isolated effects, clusters of histidines such as His93^(2.63)^, His172^(4.64)^, His178^(*ECL*2)^, and His296^(6.58)^, (The superscript uses the Ballesteros–Weinstein numbering.^65,66^ In this notation, the first number indicates the transmembrane helix, and the number after the dot marks the residue’s relative position within that helix.) which showed coordinated changes in protonation between pH:6.5 and pH:8.0. This pattern indicates that ECLs may act as pH-sensitive modules, potentially modulating extracellular gating and ligand-binding interactions, in agreement with previous observations that histidine-rich extracellular regions can tune receptor sensitivity to pH.^67–69^ In comparison, several intracellular loop (ICL) residues (e.g., His241^(*ICL*3)^, His256^(*ICL*3)^, and Glu338^(8.56)^) also exhibited pH-dependent variability, although to a more moderate degree, suggesting an auxiliary but less dominant role in intracellular regulation. As expected, residues with intrinsic p*K*_*a*_ values outside the studied range (e.g., Lys305^(7.31)^, Cys190^(*ECL*2)^, and Glu122^(3.41)^) remained largely stable, underscoring that the dynamic protonation landscape is primarily driven by histidines.

An electrostatic modulation was also reflected in Na^+^ distributions: across all pH conditions and replicas, Na^+^ remained consistently absent from the canonical Asp79^(2.50)^ site while preferentially localized near Asp113^(3.32)^ (Supporting Table S5). This observation parallels the mechanism proposed by Ballabio and Capelli (2025),^70^ who showed that β_2_AR inactivation can proceed without stable Asp79^(2.50)^ Na^+^ binding, further supporting the idea that protonation-dependent microenvironments shape allosteric ionic accessibility.

To assess the structural consequences of these protonation-driven changes, we analyzed the root mean squared fluctuation (RMSF) (Figure 2A,B). Although the average deviations appear moderate in Figure 2A, the ICL3 region in the Control run shows a sharp peak in flexibility, particularly in its core segment (residues 241^(*ICL*3)^–260^(*ICL*3)^), reaching approximately 14 Å. This central flexibility was attenuated under all CpHMD conditions, suggesting stabilization by pH-dependent protonation. In contrast, the ICL3 edge regions (residues 230^(5.63)^–240^(*ICL*3)^ and 261^(*ICL*3)^–266^(6.28)^) are more stable in the Control compared to CpHMD runs. Consistent with prior work, the elevated mobility of ICL3 reflects its intrinsic disorder in inactive class A GPCRs rather than insufficient equilibration.^50,51,57,71^ Moreover, ECL2 displays minimal fluctuation in Control but is more flexible in CpHMD runs, pointing toward a dynamic coupling between ECL2 and ICL3 modulated by pH-sensitive ionization (Figure 2B).

**Figure 2:**
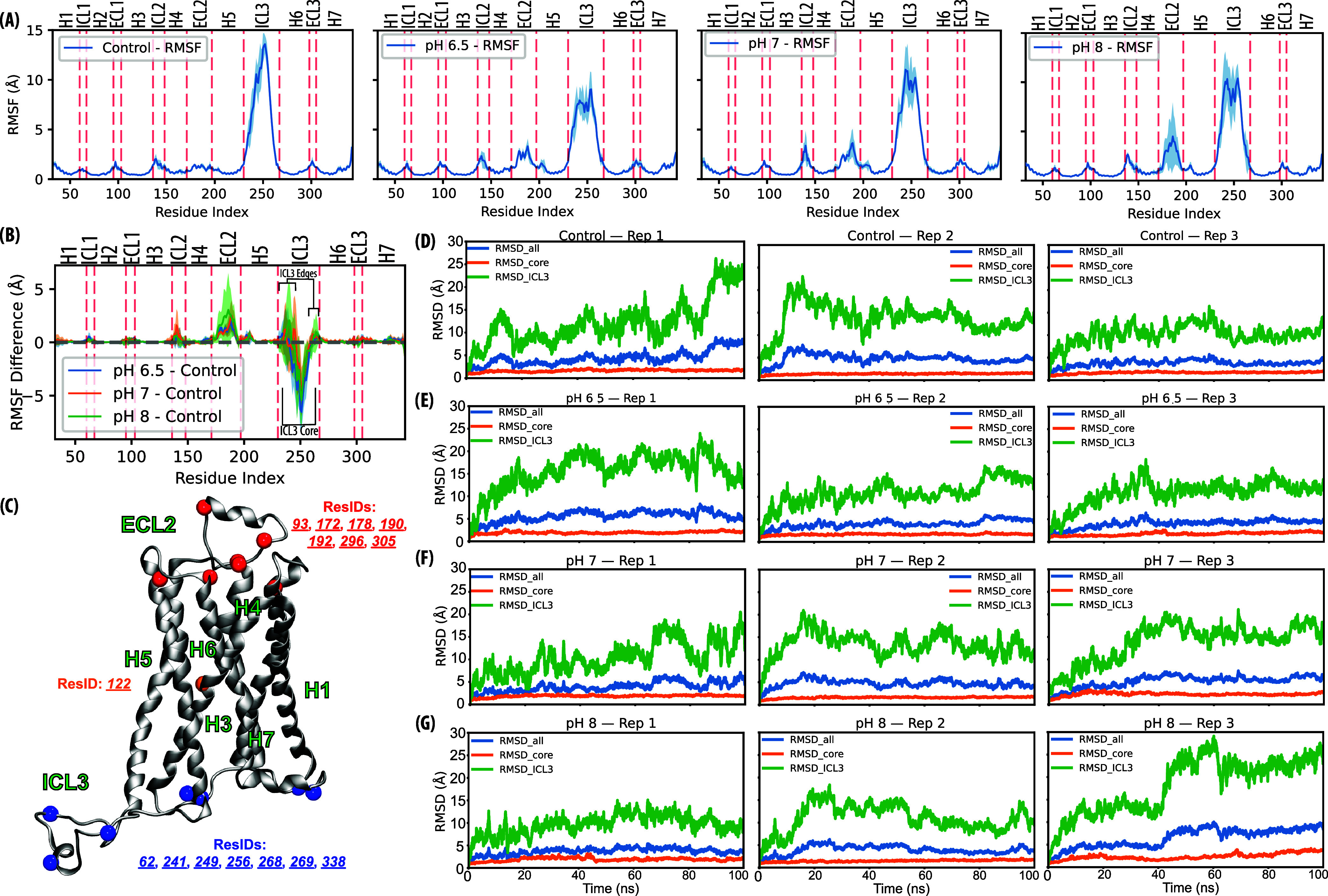
Global fluctuations and dynamics of inactive β_**2**_AR. (A) RMSFs of the four conditions. Blue shading indicates the RMSF deviation. (B) RMSF differences of CpHMD runs with respect to the Control runs. ICL2, ECL2, and ICL3 edges show increases, whereas the ICL3 core shows a significant decrease in fluctuation. (C) Representation of the location of ionizable residues those changed their protonation states throughout the trajectory, colored as red (extracellular), orange (transmembrane), and blue (intracellular) surfaces. (D–G) RMSD analysis compares the deviations in the entire protein (blue), core (no ICL3, orange), and ICL3 (green), in three replicates of (D) Control, (E) pH:6.5, (F) pH:7.0, and (G) pH:8.0, revealing that ICL3 significantly contributes to the overall structural deviation under varying pH conditions. Core trajectory, on the other hand, is relatively stable.

Further support for these trends was provided by differential RMSF analysis using Control as a reference (Figure 2B). The highest ΔRMSF values were observed in ICL3, where deviations between the Control and pH:6.5 simulations exceeded 6 Å. Interestingly, atomic fluctuations in the remaining loop regions, particularly in ECL2, were elevated at pH:8.0. This suggests a potential trade-off mechanism, where the reduced mobility of ICL3 may be compensated for by increased flexibility in surrounding loop regions. This also supports the reported allosteric coupling mechanism between ICL3 and ECL2,^51^ which is essential to regulate receptor dynamics. Notably, CpHMD simulations captured the dynamics of ICL3, revealing nearly a 50% reduction in its mobility. This emphasizes the possible role of protonation-state changes in modulating loop flexibility.

Although ECL2 does not directly interact with ICL3, its conformational dynamics can influence the movement of TM6, thus modulating the position of ICL3, supporting the notion of mechanical correspondence between these regions. This observation is consistent with structural and functional studies^72,73^ that identify ECL2 as an allosteric exosite that not only contributes to ligand engagement but also modulates intracellular loop dynamics by coupling between transmembrane helices. This coupling is further supported by the spatial distribution of titratable residues (Table 1), many of which are located in ECL2 and ICL3 (Figure 2C).

The influence of changes in protonation state on structural dynamics is observed in root-mean-square deviation (RMSD) trajectories (Figure 2D–G). Each condition was analyzed with three independent replicates, revealing reproducible yet distinct conformational behaviors among the All, Core (excluding ICL3), and ICL3 regions. Although the Core region maintained relatively stable RMSDs throughout the trajectories, indicating global equilibrium, ICL3 consistently exhibited the greatest conformational variability across replicates (Control: 12.03 ± 2.66 Å; pH:6.5: 12.45 ± 2.52 Å; pH:7.0: 12.51 ± 2.68 Å; pH:8.0: 12.91 ± 4.27 Å). In particular, ICL3 in the Control displayed early sharp deviations, whereas at pH:8.0, larger drifts emerged at later stages, indicating delayed conformational rearrangements. The replicate-wise representation underscores that these fluctuations are not isolated events but recurring features of ICL3 dynamics under varying protonation environments. Given the established role of ICL3 flexibility in the coupling of G-proteins and the propagation of intracellular–extracellular signal,^50,74,75^ the observed replicate-consistent conformational heterogeneity suggests that pH-dependent protonation shifts can modulate functionally relevant states of β_2_AR.

### pH-Driven Variations in Collective Motions

Given the fluctuations across pH and the ionization state changes in critical loop residues, we investigated whether pH variations drive global structural changes and modulate the receptor’s conformational states. Structural and dynamic changes in protein trajectories across four conditions highlight RMSF profiles along the first principal component (PC1)^52^ (Supporting Figure S3A). Here, PC1 prominently captures the dynamics of flexible regions such as ICL2, ICL3, and ECL2. Among these, ICL3 stands out with the most pronounced dynamics under all conditions.

The cumulative variance plot shows that the first five principal components (PC1–PC5) account for approximately 72–84% of total variance in the conformational space and were used to compute RMSIP values, which quantify the similarity of dominant motions across trajectories (Supporting Figure S3B,C). The highest RMSIP values were observed between replicate runs for both Control (0.74–0.78) and pH:7.0 (0.60–0.76), indicating consistent dynamic behavior. In contrast, pH:6.5 exhibited the lowest similarity between runs (0.52–0.62), suggesting greater variability under acidic conditions. Among different conditions, the greatest overlap in principal component modes was observed between Control and pH:7.0 (up to 0.79), whereas pH:6.5 and pH:8.0 shared weaker overlaps with other states, reflecting their pH-dependent distinct dynamic profiles.

To characterize loop mobility along the dominant mode of motion, trajectories from each pH condition were projected onto PC1, and RMSF values were recalculated accordingly (Figure 3). We focused on ICL2, ICL3, and ECL2, since they greatly contributed to global variance (Supporting Figure S3A). For ICL2, the Control run exhibited the lowest RMSF, while CpHMD conditions showed increased fluctuations. The highest RMSF values were observed at pH:7.0, followed by pH:8.0, which suggests increased conformational plasticity within the inactive ensemble, potentially enabling local rearrangements at the intracellular interface without full activation (Figure 3A).

**Figure 3:**
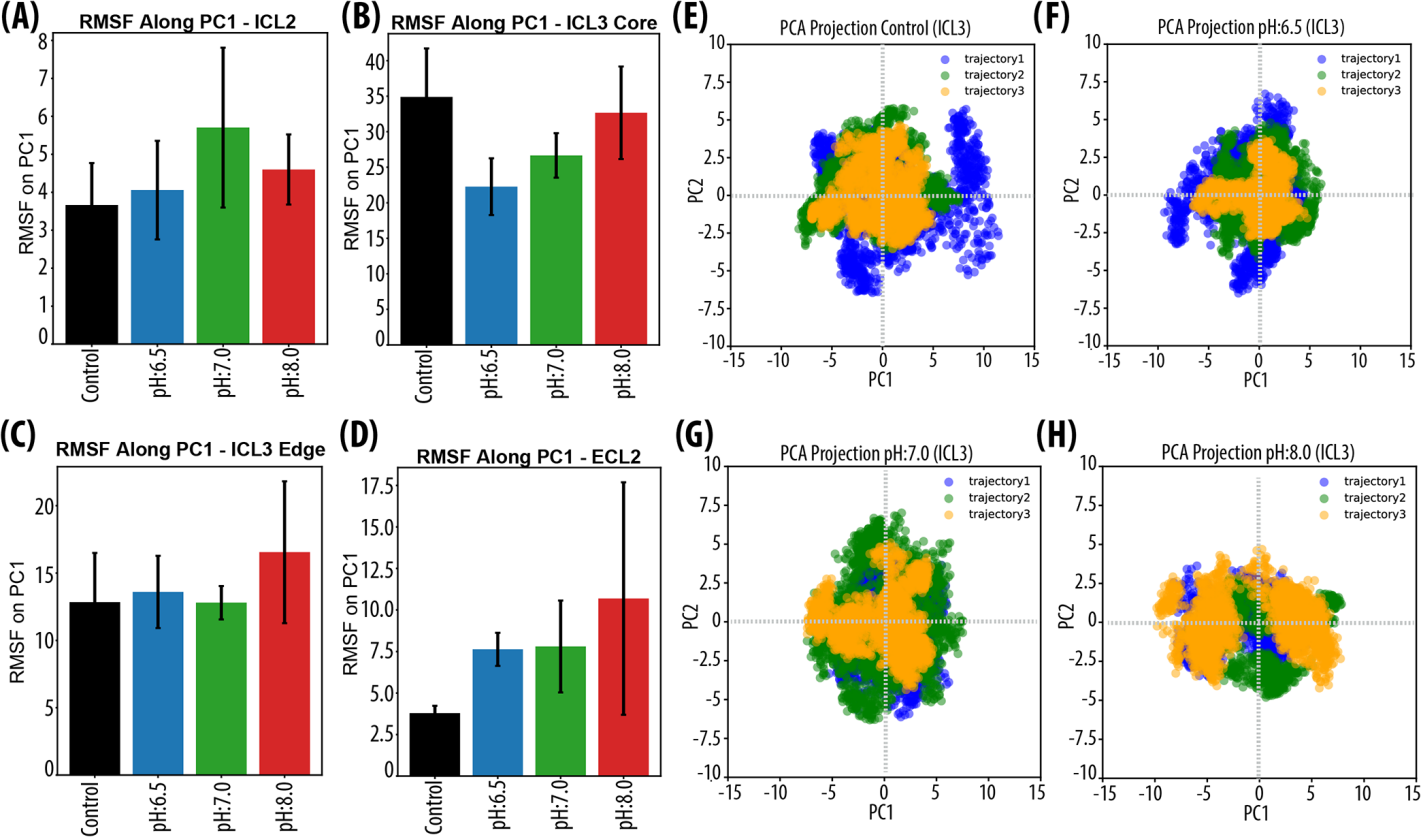
Structural and dynamic analysis of the inactive β_2_AR ensemble under different pH conditions. RMSF projected onto PC1 to capture greater conformational flexibility across conditions for (A) ICL2, (B) ICL3 Core, (C) ICL3 Edge, and (D) ECL2. Control is black, pH:6.5 is blue, pH:7.0 is green, and pH:8.0 is red. (E–H) PCA scatter plot to visualize how the trajectory replicates separated ICL3 dynamics along the two most significant directions of variance (PC1, PC2). Blue, green, and orange colors indicate trajectories 1, 2, and 3, respectively.

Similarly, ICL3 and ECL2, the largest loop regions of the receptor, exhibited different mobility profiles in CpHMD runs compared to the Control. This was possibly due to the abundance of ionizable residues in the loop regions, which altered their protonation states over the course of the CpHMD simulations. As given in Figure 3B, the core region of ICL3 was most stable under acidic conditions (pH:6.5). At physiological pH, both His241^(*ICL*3)^ and His256^(*ICL*3)^ remained largely protonated (+1), whereas Glu249^(*ICL*3)^ was predominantly neutral, generating a balanced electrostatic environment that favored intraloop stabilization. As pH increased, histidines lost their positive charge, and Glu249^(*ICL*3)^ became increasingly deprotonated (−1), disrupting this balance and producing greater conformational fluctuations. Conversely, in the Control condition, the lack of these protonation-dependent interactions led to increased flexibility. At the edges of ICL3, mobility was higher, especially at pH:8.0 and at pH:6.5 compared to Control and pH:7.0, suggesting a pH-dependent redistribution of flexibility between the loop core and its termini (Figure 3C). In the Control simulations, the lack of titratable residues at the ICL3 edges and the absence of protonation-driven electrostatic rearrangements likely restricted local flexibility, resulting in a more mechanically constrained conformation that is less responsive to structural perturbations originating from the loop core (Figure 3C).

Finally, ECL2 reflected the effects of pH-dependent protonation changes with progressively increased mobility (Figure 3D). In the Control run, RMSF remained low (∼3 Å), whereas fluctuations increased with pH, reaching ∼10 Å at pH:8.0. This trend aligns with the gradual transition of His172^(4.64)^ and His178^(*ECL*2)^ from their protonated (+1) to neutral (0) states. At low pH, their positive charge stabilizes electrostatic contacts within ECL2 and between ECL2 and adjacent helices. At higher pH, the loss of these stabilizing interactions permits greater loop flexibility and transient extracellular opening. To further characterize the conformational behavior of ICL3, the trajectories were projected onto PC1–PC2 subspace (Figure 3E–3H). In Control, the motion of the ICL3 core dominated the overall loop dynamics. In trajectory 1, the conformational space covered a broad region (PC1: −7.5 to 12; PC2: −6 to 6), indicating substantial internal flexibility and dynamic reorganization within the loop. Trajectories 2 and 3 sampled narrower regions (PC1: −7.5 to 7.5; PC2: −3 to 6), suggesting more constrained dynamics or repeated sampling of preferred conformations (Figure 3E).

Under mildly acidic conditions (pH:6.5), the conformational space along PC1 narrowed markedly (−9 to 5) while PC2 remained similar (−6 to 6). This restriction corresponds to a stabilized ICL3 core, where His241^(*ICL*3)^ and His256^(*ICL*3)^ are predominantly protonated, and Glu249^(*ICL*3)^ remains largely neutral (Table 1). The combined effect of these protonation states produces an electrostatically balanced microenvironment that minimizes the intraloop repulsion and limits the structural variability (Figure 3F).

At physiological pH (pH:7.0), the PC1 range remained confined (−6 to 6), while PC2 broadened (−7 to 7), consistent with partial deprotonation of His241^(*ICL*3)^ and His256^(*ICL*3)^ from +1 to neutral. This reduction in positive charge facilitates subtle out-of-plane motions while maintaining the global stability of the loop scaffold (Figure 3G).

Under basic conditions (pH:8.0), the conformational landscape expanded further (PC1: −9 to 9), and multiple metastable substates appeared. This behavior reflects the complete neutralization of His241^(*ICL*3)^ and His256^(*ICL*3)^, together with increased deprotonation of Glu249^(*ICL*3)^ to its negatively charged state. The resulting change in electrostatic balance enhances intraloop repulsion and drives broader rearrangements, while PC2 fluctuations remain comparatively limited (from −5 to 4.5) (Figure 3H).

Together, these results demonstrate that the protonation balance among His241^(*ICL*3)^, Glu249^(*ICL*3)^, and His256^(*ICL*3)^ defines the electrostatic landscape of ICL3, shifting from a stabilized, compact configuration under acidic conditions to a more dynamic and heterogeneous ensemble at neutral and basic pH. In line with the overall pH-dependent dynamics of β_2_AR, this pattern highlights that while the ICL3 core remains electrostatically stabilized under acidic conditions, the surrounding loop regions gain flexibility as pH increases. Such differential behavior suggests that intracellular and extracellular loops collectively function as dynamic modulators, fine-tuning the receptor’s conformational adaptability and potentially influencing its transition between functional states under varying physiological pH conditions.

### CpHMD Reveals pH-Dependent Adjustments in the Hydrogen-Bond Network of Loop Regions

Hydrophilic residues, unlike their hydrophobic counterparts, are more likely to form salt bridges and hydrogen bonds, which are key interactions that contribute to proper folding and function of the receptor.^76^ Therefore, we analyzed the hydrogen -bonding patterns of 14 titratable residues (excluding Cys190 due to its minimal protonation change; Figure 4A,B) and found that the number of hydrogen-bond donors in the Control was consistently lower than in the CpHMD runs. In contrast, the number of hydrogen-bond acceptors remained largely comparable, except for Glu249^(*ICL*3)^ and His256^(*ICL*3)^, which acted as strong acceptors. Notably, Glu249^(*ICL*3)^ formed more than 60 hydrogen bonds during the Control simulations, indicating its central role in stabilizing local electrostatic networks within the ICL3 region. These results suggest that under dynamic protonation conditions, hydrogen-bonding networks become more adaptable and responsive to the local environment, allowing the receptor to fine-tune its conformational stability in a pH-dependent manner, a flexibility that is inherently restricted in conventional MD simulations.

**Figure 4:**
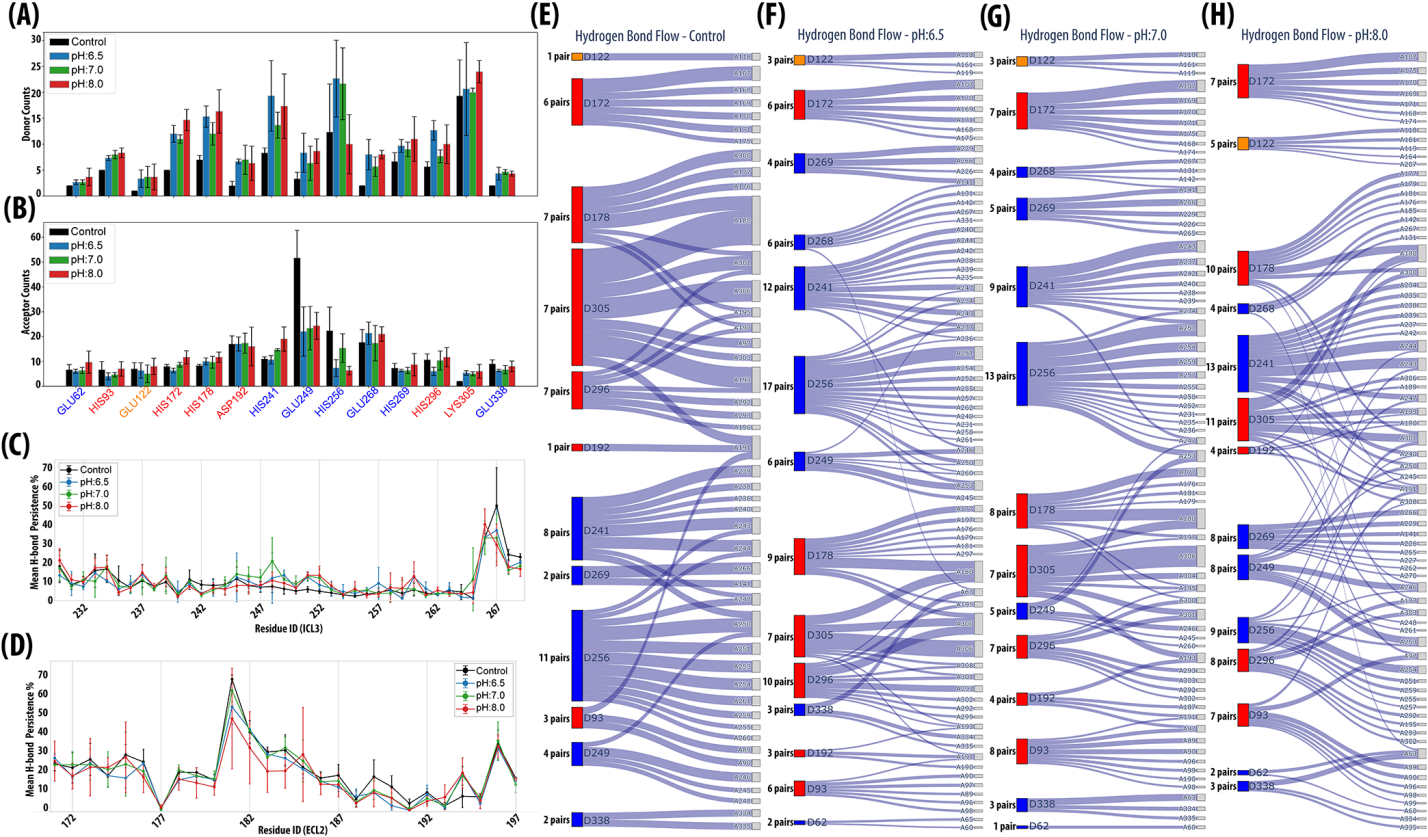
Hydrogen-bond persistence and titratable residue interactions across pH conditions. (A, B) The number of hydrogen bonds varies with pH when 14 titratable residues (excluding Cys190^(*ECL*2)^) are concerned. The Control condition has fewer hydrogen donors/acceptors, except for Glu249^(*ICL*3)^, suggesting that pH fluctuations allow residues to reorganize bonding interactions, unlike classical MD, which imposes rigid ionization constraints. Orange is TM residue, blues are ICL residues, and reds are ECL residues. Black bars are Control, blue bars are pH:6.5, green bars are pH:7.0, and red bars are pH:8.0. (C) Hydrogen bond persistence of ICL3 residues represent that Control run has lower bond persistence, especially in the ICL3 core. (D) Hydrogen bond persistence of ECL2 increases in the Control run. The persistence decreases at pH:8.0. (E–H) Individual Sankey diagrams for key residues acting as hydrogen bond donors. Each panel depicts the hydrogen bond interaction flow under (E) Control, (F) pH:6.5, (G) pH:7.0, and (H) pH:8.0 conditions. Ionizable residues are colored by their structural domain: red (ECL), blue (ICL), and orange (TM), while gray nodes represent nonionizable residues involved in hydrogen bonding. Boxes denote observed hydrogen bonds with box thickness scaled to the average number of hydrogen bonds formed between residue pairs across trajectories.

The hydrogen bonds were further explored in ICL3 and ECL2 to understand how persistent these interactions remained throughout the trajectory. Figure 4C shows that hydrogen bonds within ICL3 (residues 230^(5.69)^–269^(6.31)^) were weakly persistent in the Control run, particularly in the core segment (residues 246^(*ICL*3)^–262^(6.24)^). This is in accordance with the higher mobility of the ICL3 core region observed in Control runs with respect to CpHMD runs, Figure 2D. In contrast, ICL3 residues showed more persistent hydrogen bonds at all pH conditions compared to the Control state. ECL2, on the other hand, shows completely opposite behavior, indicating an existing correspondence between the number of persistent hydrogen bonds and the mobility of ECL2. Especially, the number of hydrogen bonds for residue range 181^(*ECL*2)^-192^(45.51)^ was the lowest at pH:8.0, explaining the highest ECL2 mobility (Figures 4D and S4–S6).

Moreover, the ionizable residues and their interaction partners show that the hydrogen bond flow was also affected by the protonation state changes. In the network shown in Figure 4E–H, red, blue, and orange boxes represent ionizable residues located in the ECL, ICL, and TM domains, respectively. Gray boxes denote other residues involved in hydrogen bonds with edge thickness proportional to the average number of hydrogen bonds formed between the corresponding residues across trajectories.

Domain-level analysis of recurrent hydrogen-bonding interactions, ordered by donor and acceptor residue IDs, revealed distinct pH-dependent patterns (Figure 4E–H). In ICLs, donor activity and bonded pairs were minimal in the Control, with a total of five residues, His241^(*ICL*3)^, Glu249^(*ICL*3)^, His256^(*ICL*3)^, His269^(6.31)^, and Glu338^(8.56)^, showing only a few yet persistent interactions (n = 8, 4, 11, 2, 2, respectively) (Figure 4E). In CpHMD runs, two additional residues, Glu62^(12.48)^ and Glu268^(6.30)^, in the ICL regions appeared as donors. At pH:6.5, the ICLs showed strikingly new hydrogen bond formations with a variety of residues: His241^(*ICL*3)^ formed hydrogen bonds with 12 unique acceptors, His256^(*ICL*3)^ with 17, Glu249^(*ICL*3)^ with 6, Glu268^(6.30)^ with 6, and His269^(6.31)^ with 4 hydrogen bonds (Figure 4F). This nearly 50% increase in hydrogen bonding suggests protonation-induced rewiring of intracellular networks. Moreover, although donor activity and variability were slightly lower at pH:7.0 compared to pH:6.5, it remained elevated compared to Control (Figure 4G). Based on five residues common between the Control and pH:7.0, pair variability increased by 30%. At pH:8.0, it increased again by ≈ 50% (Figure 4H).

The residues of the ECLs (His93^(2.63)^, His172^(4.64)^, His178^(*ECL*2)^, Asp192^(45.51)^, His296^(6.58)^, and Lys305^(7.31)^) formed more variable hydrogen bonds as pH increased. ECL donors exhibited limited but persistent interactions in Control (Figure 4E), but pH:6.5 initiated a pronounced increase in pair variability: His93^(2.63)^ (6 acceptor pairs), His172^(4.64)^ (6), His178^(*ECL*2)^ (9), Asp192^(45.51)^ (3), His296^(6.58)^ (10), Lys305^(7.31)^ (7) and overall were ≈ 30% higher compared to the Control (Figure 4F). This trend continued until pH:7.0 (≈ 30% more) (Figure 4G) and peaked at pH:8.0 (≈ 50% higher) (Figure 4H), where His178^(*ECL*2)^ and Lys305^(7.31)^ formed hydrogen bonds with 10 and 11 unique acceptors, respectively. His93^(2.63)^, nearly silent in the Control, became notably active at pH:8.0 (7 acceptor pairs), reflecting a pH-induced remodeling of the extracellular surface. This growing diversity of hydrogen-bond partners at higher pH values indicates increased structural plasticity in the extracellular loops, which may modulate the accessibility and dynamics of the ligand-binding pocket.

Glu122^(3.41)^ in the transmembrane domain exhibited a steady increase in hydrogen bonding from Control (1 pair) (Figure 4E) to pH:8.0 (5 pairs) (Figure 4H), suggesting a pH-responsive role rather than a strictly constant structural scaffold.

These findings indicate that pH exerts a dual regulatory influence on the receptor’s hydrogen-bonding landscape. Under acidic conditions, increased hydrogen-bond diversity within intracellular loops, particularly ICL3, suggests a shift toward inward-facing conformations that may prime the receptor for G-protein coupling. Conversely, at basic pH, enhanced hydrogen bonding among extracellular residues, especially within ECL2, points to greater structural flexibility that could facilitate ligand entry or stabilization of preactive states. In contrast, Control simulations with fixed protonation states exhibited minimal bonding dynamics, emphasizing that protonation flexibility is essential to reveal the full range of conformational adaptability and functional coupling in β_2_AR.

### Microswitch Analysis Confirms Inactive-State Sampling across All pH Conditions

To assess whether the receptor sampled inactive-like conformations, we monitored five canonical GPCR microswitches. The ionic lock distance between Arg131^(3.50)^ and Leu268^(6.30)^ reports on TM3–TM6 coupling and reflects the stability of the Arg131^(3.50)^–Glu268^(6.30)^ salt bridge, which is below 10.5 Å in the inactive state of the receptor. The Tyr219^(5.58)^Cζ –Tyr326^(7.53)^Cζ distance captures the opening of the extracellular aromatic gate and is expected to be above 14.6 Å in the inactive state. The NPxxY RMSD (Asn322^(7.49)^–Cys327^(7.54)^) after alignment to the inactive 2RH1 structure tracks the characteristic inward rotation of TM7, and should be below 2 Å in the inactive state, while the PIF RMSD reflects rearrangements of the Ile121^(3.40)^–Phe282^(6.44)^ pair linked to TM3/TM6 repacking needs to be below 2.2 Å in the inactive state.^70^

Across all pH conditions, the microswitch readouts remained largely consistent with an inactive-state ensemble, with per-trajectory distributions revealing modest pH-dependent modulations rather than activation-like transitions. NPxxY RMSD values (Figure 5A) stayed well below the 2 Å inactive threshold, showing narrow distributions for each replica, indicating that the intracellular portion of TM7 does not undergo the characteristic outward twist associated with activation. Similarly, the Arg131^(3.50)^–Glu268^(6.30)^ ionic-lock distance (Figure 5B) remained centered below 10–11 Å in all trajectories, consistent with an intact TM3 to TM6 coupling interface range associated with ionic-lock rupture in active GPCRs.

**Figure 5:**
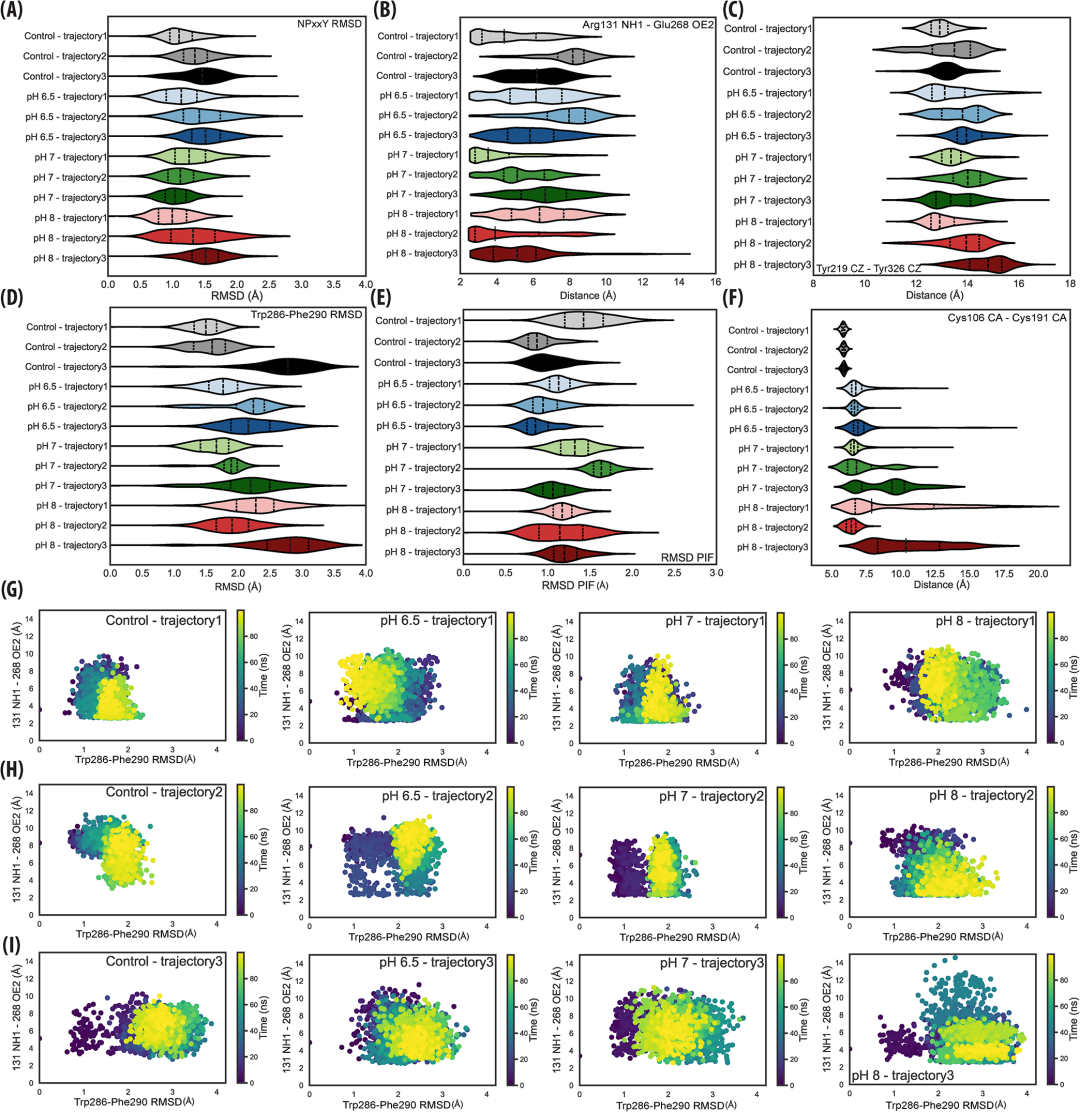
Canonical GPCR microswitch analysis across CpHMD trajectories. (A) NPxxY motif RMSD (N322^(7.49)^–C327^(7.54)^) after alignment to the inactive 2RH1 structure; values remain below the ∼2 Å threshold expected for inactive conformations. (B) Ionic-lock distance between Arg131^(3.50)^–NH1 and Glu268^(6.30)^–OE2, which typically stays below 10 Å in inactive GPCRs. (C) Extracellular aromatic-gate distance (Tyr219^(5.58)^–Tyr326^(7.53)^ Cζ), expected to remain >14.6 Å in the inactive state. (D) Rotamer toggle-switch RMSD for the Trp286^(6.48)^–Phe290^(6.52)^ pair, reporting on local rearrangements around TM6. (E) PIF motif RMSD (Ile121^(3.40)^–Phe282^(6.44)^) relative to the inactive 2RH1 reference; values ≤2.2 Å indicate an inactive configuration. (F) Cys106^(3.25)^–Cys191^(45.50)^ Cα–Cα distance; the disulfide linkage was fixed via DISU patches, so variations reflect geometric strain. Gray scale: Control, blue scale: pH:6.5, green scale: pH:7.0, red scale: pH:8.0. Thick dashed lines represent the median, and thin dashed lines are IQRs. (G–I**)** Scatterplots of Arg131^(3.50)^–Glu268^(6.30)^ distance versus Trp286^(6.48)^–Phe290^(6.52)^ RMSD for trajectories 1–3, respectively, illustrating trajectory-specific sampling within the inactive conformational landscape. Color bar represents the trajectory time in nanoseconds.

The extracellular Y–Y gate (Tyr219^(5.58)^ Cζ – Tyr326^(7.53)^ Cζ distance) also remained strictly within the inactive regime (Figure 5C): all trajectories maintained values above 14.6 Å, with no excursions toward the ≤ 8 Å closure characteristic of active-like gating. This stability was mirrored at the level of the rotameric microswitches. Both the Trp286^(6.48)^–Phe290^(6.52)^ toggle switch RMSD (Figure 5D) and PIF motif RMSD (Figure 5E) exhibited compact distributions across pH conditions. The Cys106^(3.25)^–Cys191^(45.50)^ disulfide bridge exhibited modest pH-dependent geometric fluctuations (Figure 5F), consistent with a preserved covalent linkage. Because disulfide connectivity was explicitly constrained in the simulation topology, the observed distance variations reflect local conformational strain. Finally, scatterplots of ionic-lock distance versus toggle-switch RMSD for each trajectory (Figure 5G-I) showed no active-like patterns, instead forming tight inactive-state clouds across all replicas. Collectively, these microswitch analyses demonstrate that all CpHMD trajectories remain within the inactive landscape and that pH primarily modulates local flexibility rather than driving transitions toward activation.

### Mutual Information Analysis Reveals Increased Dynamic Communication between Intra- and Extracellular Loops in CpHMD

Mutual information (MI) is a powerful tool for uncovering how different parts of a protein communicate by capturing linear and nonlinear dependencies in their movements, which makes it especially useful for studying how structural changes propagate across a protein.^56^ Our previous studies have shown that MI effectively highlights key residues involved in information transfer and helps explain how dynamic coupling influences protein stability and function.^56,57^ Building on this, we used MI to explore how intra- and extracellular domains exchanged dynamic information under different pH conditions in CpHMD simulations and whether the change in the protonation state would eventually have an impact on information transfer.

Our initial MI analysis was based on Cα positional fluctuations. The pairwise MI was plotted across conditions (Figure 6A) and overlaid with contact maps (*d*_*i*,*j*_ ≤ 6 Å) to identify the regions with the highest levels of information sharing between distant pairs without contact. Because a 6 Å heavy-atom cutoff is broadly accepted for defining residue contacts in protein structural analyses,^77–79^ this threshold allows direct coupling to be separated from long-range information transfer without biasing the comparison across pH conditions. These heatmaps exposed how CpHMD increased long-range pairwise interactions in different domains, especially the interactions within ICL3 and between ECL2 and ICL3, which are most visible under pH:8.0 conditions. However, in the Control, the information sharing within the loops appeared to be more localized and confined (Figure 6A).

**Figure 6:**
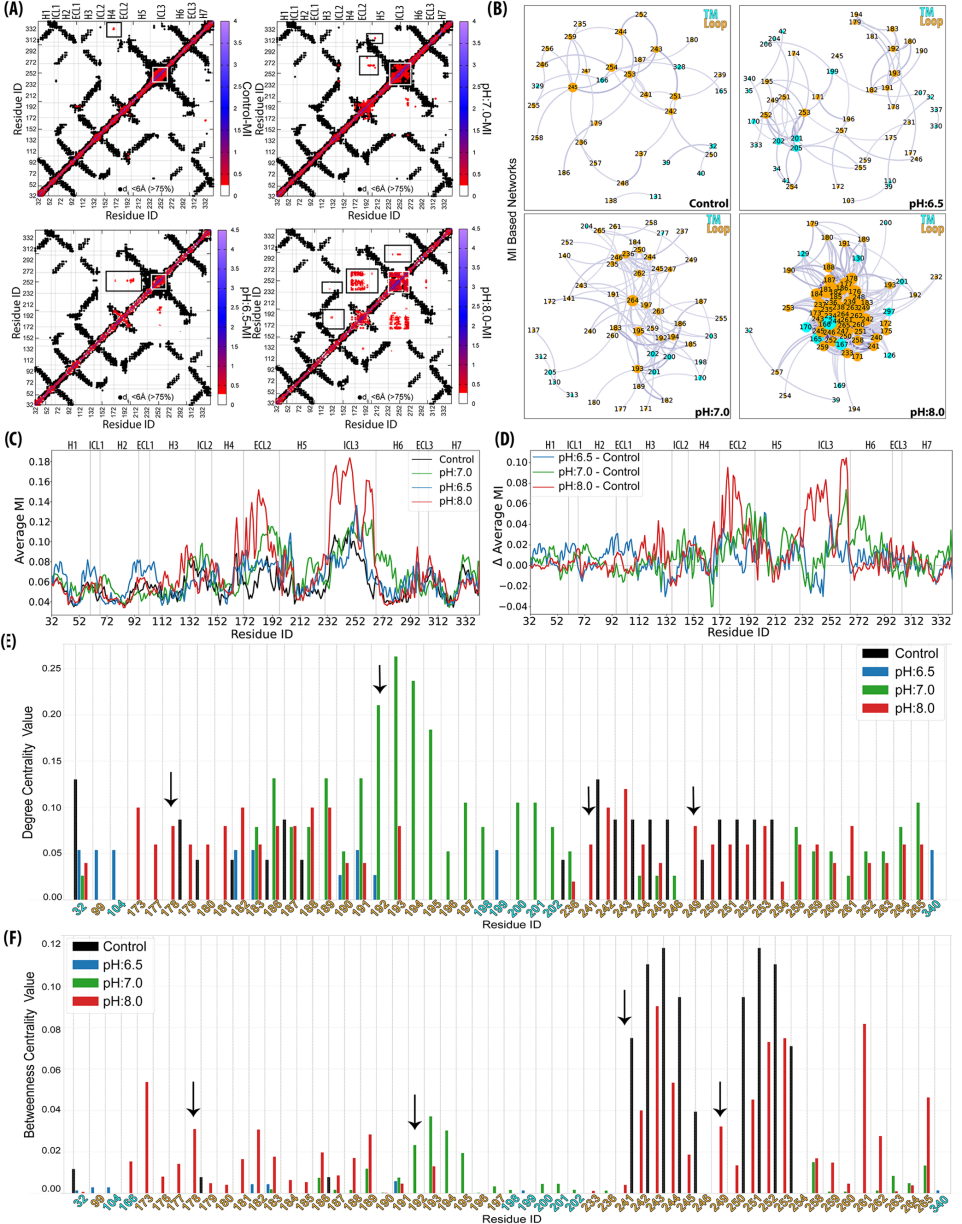
MI-based interaction network analysis of molecular dynamics simulations focusing on Cα atoms. (A) Pairwise MI heatmaps (MI ≥ 0.25) across Control, pH:6.5, pH:7.0, and pH:8.0 illustrate how pH changes increase long-range interactions, particularly within ICL2, ECL2, and ICL3. The MI values were overlaid with the contact map where residue pairs are considered as in contact if they are within 6.00 Å at least 75% of the trajectory. (B) MI-based interaction networks were generated by plotting only MI ≥ 0.25 interactions. Loops (orange nodes) consistently exhibit higher degree centrality, emphasizing their role as key hubs in information transfer. While fewer helical residues (blue) contribute to information sharing, those that do tend to localize at the loop edges. Compared to the Control condition, the CpHMD runs exhibit reorganized network topologies, with notable shifts in connectivity patterns. pH:7.0 and pH:8.0 conditions display the most structured connectivity, where ECL2 and ICL3 emerge as dominant domains for centralities. (C) Average MI and (D) differences in average MIs of CpHMD runs with respect to the Control run. (E) Residue-wise degree centrality and (F) betweenness centrality across four conditions (Control (black), pH:6.5 (blue), pH:7.0 (green), and pH:8.0 (red)) provide insights into the topological significance of residues in the MI-based interaction networks. Degree centrality highlights residues with high connectivity, where ECL2 shows increased centrality at pH:7.0, suggesting its role as key mediators of localized interactions. Stronger long-range centralities are particularly evident at ECL2 and ICL3 at pH:7.0, Control, and pH:8.0, indicating condition-dependent connectivity changes.

Furthermore, the analysis of the residue interaction network, which was based on MI values (as mentioned in the Methods section), was created for residue pairs with *MI* ≥ 0.25 to reveal the long-range residue pairs sharing at least a quarter bit of mutual information and where short-range interactions (residues within 6 positions)^56^ were excluded (Figures 6B and S7–S10). Interestingly, each simulation condition displayed a distinct pattern in the connectivity between helices and loop regions, where loops had the largest degree of centrality under all conditions. Overall, fewer helical residues contributed to the information sharing, and those exhibiting higher connectivity were located close to the loop regions. In contrast, loop residues, despite their inherent flexibility, appeared as key information hubs, reinforcing their role in allosteric interactions. Notably, unlike the Control run, changes in pH conditions led to a reorganization of the network topology, with differences in edge thickness and connectivity patterns indicating pH-dependent shifts in interaction dynamics. Especially, simulations at pH:7.0 and pH:8.0 had the most organized loop connectivity patterns, where ECL2 and ICL3 were the leading domains for the centralities. At all three pH values, high MI and centrality indicate strongly coupled motion among loop regions, which likely serves to propagate signals across the receptor.

Additionally, an average MI (<MI>) for each residue was determined (Figure 6C) to highlight intercondition variations. The Control condition exhibited a relatively uniform but low baseline for <MI>. Upon pH perturbation, the helices responded with condition-specific enhancements: pH:6.5 induced a broad increase in <MI> within ECL1 and the extracellular segments of H3 and H5, consistent with the stabilization of local electrostatic interactions through protonation of titratable residues. At pH:7.0, coupling intensified in the intracellular regions of H5, H6, and H7, as well as ECL2 and ICL2, reflecting increased communication across intracellular–extracellular interfaces. Notably, the highest <MI> values emerged under basic conditions (pH:8.0), especially within ECL2, ICL3, and the intracellular portion of H3, indicating enhanced long-range coordination among loop domains. This increase is consistent with the pH-dependent loss of positive charge from histidine residues and the higher prevalence of deprotonated acidic residues, both of which reshape local electrostatic profiles and broaden the conformational communication network. Such pH-driven rearrangements resemble the protonation-coupled conformational switches reported for rhodopsin^80^ and the agonist-induced flexibility observed in the β_2_AR coupling domain,^12^ highlighting the shared mechanistic basis between protonation-dependent structural dynamics and activation-related transitions.

Difference <MI> plots (ΔMI) in Figure 6D suggest that ECL2 and ICL3 are the the main pH sensors, particularly at pH:8.0, showing the highest increase in ΔMI, while H1, H2, and H4 remained largely invariant. Those significant increases in ECL2 and ICL3 clearly indicate enhanced allosteric responsiveness and shifts in flexibility, followed by structural rearrangements. Furthermore, these findings are consistent with our previous observations of increased persistence and diversity of hydrogen bonds in ECL2 and ICL3 under acidic conditions (Figure 4C,D), suggesting that both ECL2 and ICL3 can act as key pH-sensitive regulatory nodes that facilitate signal transduction by modulating interdomain coordination.

Additionally, Figure 6E,F shows degree and betweenness centralities within MI-based interaction networks, highlighting the topological prominence of residues under different pH conditions. At pH:7.0, degree centrality showed a pronounced peak in ECL2 (residues 191–195), the adjacent H5 region, and the terminal part of ICL3 near H6. These clusters acted as communication nodes linking the extracellular and intracellular domains. In the Control, however, centrality was mainly concentrated in mid-ECL2 and the first half of ICL3, consistent with a more static communication network observed under fixed-protonation conditions. At pH:8.0, the entire ECL2 and ICL3 became highly connected hubs, supported by strong MI correspondence between the two loops, while lowering the pH weakened this communication: it diminished at pH:7.0 and was nearly absent at pH:6.5 (Figure 6E). This enhanced interloop coupling under basic conditions suggests a possible shift toward preactive conformations, in which extracellular and intracellular networks become more dynamically synchronized.

Betweenness centrality complemented these findings by identifying residues that mediate long-range communication. In both Control and pH:8.0, ICL3 residues exhibited high betweenness values, marking them as strategic relay points in intracellular signaling. In contrast, ECL2 showed minimal variations except at pH:8.0, where it covered the loop region and contributed to extracellular–intracellular coupling. Overall, the two centrality measures consistently emphasized the dynamic role of ECL2 and ICL3 as hubs and bridges within the receptor’s allosteric communication network, with their prominence strongly modulated by pH (Figure 6F).

### Backbone and Side-Chain Flexibility Amplifies Mutual Information in pH Adaptation

When assessing protein structure, the analysis often focuses on Cα atoms, which provide a backbone representation and are commonly used to evaluate global conformational changes. However, this backbone-centered view can be complemented by examining how dihedral angle dynamics and rotatable bonds influence receptor conformation and its response to environmental changes.^57,60,81^ To capture this additional layer of structural variability, we quantified dihedral couplings in β_2_AR using mutual information (MI). The backbone and side-chain dihedrals included in the analysis were ϕ, ψ, χ_1_, χ_2_, χ_3_, and χ_4_, with their rotameric states derived from a rotamer library.^58^

Previously, studies explored the significant role of side-chain fluctuations in the thermodynamics of ligand binding and allosteric communication within proteins. In our group, we previously demonstrated that fluctuations in side-chain rotamer states were key drivers of long-range allosteric communication within proteins, facilitating the transfer of information between distant sites.^57^ Also, Najmanovich et al. emphasized the importance of side-chain flexibility during ligand binding, noting that these conformational changes not only enable ligand accommodation but also ensure specificity and selectivity of binding sites.^81^ Additionally, Dubay et al. highlighted that significant correlations among side-chain movements can propagate structural changes across large distances within a protein, even with a fixed backbone, shedding light on the mechanics of allosteric signaling.^60^

Here, heatmap-based MI values represented the loop regions as the strongest domains of information sharing (Supporting Figure S11). Furthermore, the average MI values shared between distant residues (more than 5 residues apart) indicated that pH:6.5 displayed the highest dihedral <MI> value (*p* = 0.019), suggesting that this slightly acidic pH condition promoted the most significant coupling or information sharing among dihedral angles (Figure 7A and Supporting Table S4). On the contrary, pH:8.0 displayed the lowest dihedral <MI>, highlighting more independent internal motions and less coupled side-chain dynamics. Notably, the comparison between the Control and pH:7.0 showed no statistically significant differences, although pH:7.0 had slightly lower dihedral <MI> values than the Control. Moreover, higher dihedral <MI> values observed in the loop regions, compared to the transmembrane regions, were probably due to greater flexibility and fewer structural constraints in the loop structures (Figures 7A, S12).

**Figure 7:**
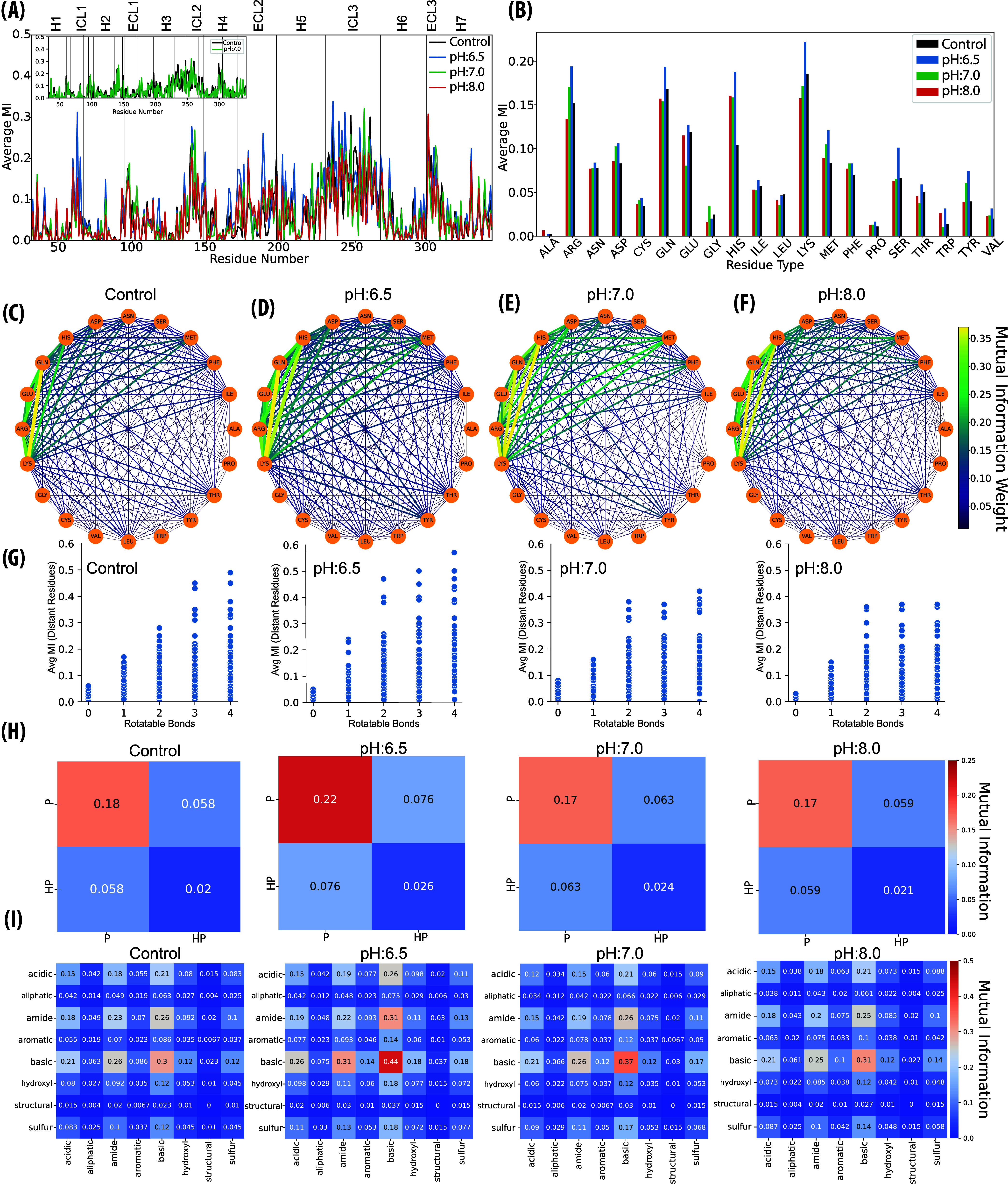
Dihedral MI analysis revealing significant insights into the side-chain dynamics and their impact on receptor structure across different pH conditions. (A) The highest average dihedral MI values are observed at pH:6.5 (*p* = 0.019), suggesting enhanced coupling among dihedral angles at this slightly acidic pH, whereas pH:8.0 exhibits the lowest MI, indicating more independent internal flexibility and less coupled side-chain dynamics at basic pH levels. Black is for Control, blue is for pH:6.5, green is for pH:7.0, and red is for pH:8.0. (B) Lysine emerges as the residue with the highest contribution to dihedral MI, followed by glutamine, arginine, and histidine, with charged residues playing a pivotal role in long-range communication and structural stability through electrostatic interactions. (C–F) MI network showing the highest shared information between lysine-glutamine, lysine-arginine, and arginine-glutamine pairs in the Control run (MI ≈ 0.45), with increased MI between lysine and glutamine at pH:6.5 (MI ≈ 0.5), suggesting enhanced residue communication at acidic conditions. Similar trends of lysine-mediated information sharing are observed at pH:7.0 and pH:8.0, with fluctuations in the strength of residue coupling, which suggests the receptor adapts dynamically to pH changes by altering its side-chain communication patterns. (G) The increase in average MI with higher numbers of rotatable bonds highlights that regions with greater flexibility contribute more to the overall communication network. (H) Polar residues, capable of forming transient interactions, show greater MI than hydrophobic residues, indicating that polar regions are more responsive to environmental changes, while the hydrophobic core remains stable. (I) Basic-basic couplings, dominated by residues such as lysine, arginine, and histidine, contribute most to the information sharing, followed by amide-basic and acidic-basic couplings, underlining the importance of electrostatic and hydrogen bond interactions in maintaining receptor dynamics across pH conditions.

Moreover, residue-based dihedral <MI> analysis revealed that Lysine has the highest contribution to average dihedral MI, followed by glutamine, arginine, and histidine (Figure 7B). Lysine, with its flexible and charged side chain, was likely involved in electrostatic interactions that could influence distant residue pairs by stabilizing various conformations through charge-based interactions. The guanidinium group of arginine and the imidazole ring of histidine are also capable of forming complex hydrogen-bond networks and electrostatic interactions, which allow them to transmit conformational changes over large distances. Furthermore, glutamine, with its polar side chain, is likely contributing through hydrogen-bonding or dipole interactions. The fact that these residues displayed high contributions to dihedral MI suggests that they were key players in maintaining the dynamic architecture of side-chain flexibility, enabling it to adapt to different conditions by facilitating structural adjustments and long-range communication.

Interestingly, histidine’s contribution to residue dihedral communication was notably lower in the Control condition than that of the pH-altered states, suggesting a restricted interaction state likely due to stable protonation and consequently a reduced dynamic rearrangement. Furthermore, we observed that pH:6.5 globally exhibited the highest dihedral <MI> across amino acids, indicating a state of enhanced all-residue dynamic coupling. The dihedrals in Control runs retained stronger MI contributions from glutamine, glutamic acid, lysine, and threonine, compared to pH:7.0 and pH:8.0, pointing to stable polar interactions under resting conditions. Meanwhile, at pH:7.0, dihedral MI increased for arginine, aspartic acid, histidine, methionine, phenylalanine, and tyrosine, indicating stronger coupling among charged and aromatic residues. This near-neutral pH may therefore optimize interaction potentials within these side chains, enhancing conformational communication relevant to activation. These findings support the idea that different pH environments tune the communication landscape of the receptor by reshaping the dynamic interplay among residue side chains.

The average dihedral MI network displayed the highest shared information, and the strength of coupling was between glutamine and lysine (MI = 0.45), which was accompanied by lysine-arginine and arginine-glutamine (MI = 0.38 and 0.35, respectively) in the Control (Figure 7C). At pH:6.5, we observed an increase in dihedral MI between lysine and glutamine (MI = 0.5), and at this pH, lysine and histidine also shared significant information (MI = 0.47). This increased dihedral MI suggested that, under slightly acidic conditions, there was an improved level of communication between specific residue dihedrals, which could contribute to more coordinated dynamics. These changes might be a direct response to the altered protonation states of the residues, affecting the overall conformation and interaction networks of the receptor (Figure 7D). At pH:7.0, shared information was observed between Lysine and three other residues (histidine, glutamine, and arginine), with comparable MI values (0.38, 0.37, and 0.37, respectively), similar to those in the Control. However, the higher number of significant lysine-centered couplings at this pH indicates enhanced electrostatic communication among positively charged and polar residues, reflecting an optimal balance between charge complementarity and conformational flexibility near physiological conditions (Figure 7E). Lastly, at pH:8.0, a further decrease in dihedral coupling led to a loss in the number of coupled residue pairs, where the strongest coupling was observed for the lysine-glutamine and lysine-histidine pairs (MI = 0.37, and 0.36, respectively) (see Figure 7F). This reduction in coupling likely reflects charge redistribution and weakened electrostatic coherence under basic conditions, which may dampen coordinated conformational transitions.

The flexibility of rotational bonds facilitates conformational changes and interactions between various structural elements, which in turn enhances the coupling and coordination of these elements.^57^ In Figure 7G, the increase in the average dihedral MI with the number of rotatable bonds indicates that regions with more rotatable bonds were consequently more involved in the overall communication network of the protein, potentially contributing to more robust and adaptive structural dynamics. Interestingly, the dihedral <MI> contributions of residues with two rotatable bonds, such as glutamine, glutamic acid, and histidine, were notably low in the Control compared to other conditions, reflecting how the absence of protonation-state changes restricts information transfer among moderately flexible side chains within the receptor.

Furthermore, polar residues, which can engage in hydrogen bonding and other electrostatic interactions, displayed a higher degree of information sharing at pH:6.5 (0.22) (Figure 7H) compared to Control (0.18), pH:7.0 (0.17), and pH:8.0 (0.17). This enhanced dihedral coupling likely arises from the ability of polar residues to form transient interactions that can readily adapt to pH-induced environmental changes, where partial protonation at a mildly acidic pH increases local polarity and strengthens hydrogen-bonding networks, thereby promoting more efficient dynamic coupling among neighboring polar residues. However, hydrophobic residues, which are typically buried within the protein core and form nonpolar interactions, contributed the least to the overall side-chain dihedral MI and indicating that the hydrophobic core remained relatively stable and less responsive to environmental changes compared to the more dynamic polar regions.

Further decomposition of dihedral-based MI based on the chemical properties of residues revealed the importance of basic–basic coupling. Basic amino acids such as lysine, arginine, and histidine are known for their ability to form strong electrostatic interactions because of their positive charge at physiological pH. The enrichment of basic–basic couplings at pH:6.5 (0.44) suggests that in this mildly acidic regime, protonation of Histidine alongside persistently charged lysine and arginine promotes transient electrostatic networks that stabilize local structure yet permit dynamic rearrangements. In all conditions, the basic–basic coupling contributed more to the information sharing, followed by the amide-basic and acidic-basic couplings, and remained highest at pH:6.5. High amide-basic and acidic-basic couplings suggested that while electrostatic interactions were paramount, hydrogen bonding (in the case of amide-basic interactions) and charge–charge interactions (in the case of acidic-basic interactions) also contributed significantly to the overall dynamics and flexibility of the protein (Figure 7I).

Overall, MI analysis based on both positional and dihedral fluctuations across different conditions demonstrated that the key communication network between the intracellular and extracellular loop regions of the receptor^50,51,72,73^ can be reliably reproduced. Incorporating variations in protonation states captured a realistic picture of the loop dynamics, which are dominant in mediating allosteric communication.

## Conclusions

This study provides a complementary perspective on the pH-dependent conformational dynamics of β_2_AR, revealing features that are not fully accessible through fixed-protonation molecular dynamics. By allowing titratable residues to exchange protons in response to local electrostatics and solvent accessibility, our CpHMD simulations uncovered conformational substates and reorganization events that suggest a fine-tuned protonation-guided modulation of receptor behavior. These effects were most pronounced within the flexible intracellular and extracellular loops, particularly ICL3 and ECL2, which harbored the majority of dynamically titrating residues.

Within this framework, our findings point to pH-dependent local adjustments that preserve the receptor’s internal mechanical balance. Under acidic conditions, histidines such as His241^(*ICL*3)^ and His256^(*ICL*3)^ remained predominantly in their protonated, positively charged states, while Glu249^(*ICL*3)^ remained largely neutral. This combination created a locally cohesive electrostatic environment that stabilized the ICL3 segment. In contrast, under basic conditions, the neutralization of histidines in ECL2 (e.g., His172^(4.64)^ and His178^(*ECL*2)^) reduced charge-based constraints, increasing ECL2 mobility and weakening hydrogen-bond persistence, consistent with its proposed role as an allosteric exosite.^63^ This reciprocal exchange of flexibility between ICL3 and ECL2 forms a “see-saw-like dynamic relationship” in which the stabilization of one loop coincides with the enhanced plasticity of the other, therefore conserving adaptability within the receptor scaffold. Such behavior is consistent with previous observations that loop elements in GPCRs play compensatory mechanical roles in maintaining receptor stability and responsiveness.^63,72^ Observed local electrostatic compensation highlights how protonation changes may be redistributed to maintain mechanical equilibrium rather than propagate large-scale structural shifts. Although these rearrangements occur within a limited temporal window, they illustrate how fine-tuned electrostatic perturbations can subtly reorganize short-range interactions, preserving conformational responsiveness across pH environments.

Our microswitch analysis confirmed that all trajectories remained within the inactive conformational ensemble across the simulated pH range. The ionic-lock distance between Arg131^(3.50)^ and Glu268^(6.30)^, the Y–Y aromatic gate separation (Tyr219^(5.58)^–Tyr326^(7.53)^), the NPxxY and PIF motif RMSDs, and the Trp286^(6.48)^–Phe290^(6.52)^ toggle-switch readouts consistently fell within the thresholds characteristic of inactive GPCRs. These results indicate that protonation-dependent effects primarily modulate local flexibility and loop coupling without driving activation-like rearrangements of the transmembrane core. Together, these findings suggest that modest pH fluctuations can fine-tune microdynamics and intrareceptor communication while preserving the overall inactive-state architecture.

Accompanying these local rearrangements was a reorganization of the hydrogen-bonding network, particularly within the loop regions. CpHMD simulations showed greater diversity in donor–acceptor pairing and redistributed hydrogen-bond persistence, most prominently in ICL3 at acidic pH and ECL2 at basic pH, indicating a dynamic adjustment of polar contacts to changing protonation states. In contrast, the fixed-protonation controls maintained a comparatively rigid hydrogen-bond pattern dominated by static acceptors, reflecting a reduced responsiveness of the loop regions. Collectively, these differences emphasize how permitting protonation flexibility can introduce additional routes for local adaptation, allowing small electrostatic shifts to modulate short-range interactions that stabilize receptor microstates.

Moreover, the analysis of MI provided additional insight into intramolecular communication within β_2_AR. Allowing titratable residues to exchange protons increased both the extent and the connectivity of dynamic correlations across the receptor. In contrast to fixed-protonation controls, which showed lower MI values and largely localized couplings, CpHMD trajectories revealed broader and more integrated communication patterns linking extracellular and intracellular loops. Regions such as ECL2 and ICL3 frequently acted as hubs of correlated motion, suggesting that changes in protonation states can modulate the efficiency and direction of internal information transfer. This enhanced dynamic connectivity under variable protonation implies that the receptor’s communication pathways may not be static, but rather adapt to electrostatic fluctuations in the environment.

Extending this analysis to side-chain dihedral angles further highlighted the importance of local flexibility in shaping these communication networks. Under fixed-protonation conditions, restricted rotamer sampling limited side-chain coupling. However, when protonation was allowed to fluctuate, residues with polar and basic side chains, particularly lysine, arginine, glutamine, and histidine, emerged as key intermediates of information transfer. Many of these residues are positioned within loop segments rich in rotatable bonds, reinforcing the view that side-chain plasticity supports adaptive communication across the receptor scaffold. Although these correlations occur on relatively short time scales, they underscore how protonation dynamics can reorganize the receptor’s internal connectivity, allowing flexible and environment-sensitive communication among structural elements.

From a functional perspective, this pH-dependent redistribution of loop flexibility may be relevant for experimentally observed pH effects on β_2_AR basal activity and coupling propensity. Although our simulations remain within an inactive microswitch signature, local changes at the loop–helix interfaces can still modulate the conformational availability of weakly G-protein-competent substates at the cytoplasmic surface. The stabilization and electrostatic tightening of ICL3 under acidic conditions can bias the geometry and accessibility of the intracellular interface that engages Gα_*s*_, consistent with reports that a lower pH enhances basal β_2_AR signaling.^12^ Mechanistically, the observed “see-saw-like” relationship can be interpreted as helix–loop coupling in which ECL2 dynamics are coordinated with the extracellular ends of TM4/TM5, while ICL3 is coupled to the intracellular ends of TM5/TM6; thus, protonation-dependent reweighting of loop electrostatics may subtly tune TM5/TM6 micropacking constraints and cytoplasmic coupling readiness without requiring activation-like rearrangements of the transmembrane core.^63,72^

In conclusion, CpHMD highlights protonation-guided reorganization of the structure, interactions, and communication in β_2_AR, offering a more nuanced view of how microscopic electrostatic events can locally shape receptor dynamics. We note that the pH range simulated here (pH:6.5–pH:8.0) corresponds to the modest microenvironmental fluctuations that β_2_AR is known to experience under physiological conditions.^12^ Within this window, our trajectories remained within the inactive basin, suggesting that such pH variations primarily fine-tune local flexibility and communication rather than driving large-scale activation on the simulated time scale.

While our results underscore the potential importance of protonation-switching residues, particularly loop histidines, as contributors to receptor flexibility and ligand accessibility, several limitations should be acknowledged. First, the trajectories were limited to 100 ns-long runs for direct comparison, where the CpHMD runs were obtained from cycles of 100 ps fixed-protonation MD sampling followed by 20 ps Monte Carlo protonation-state switching, with 1000 cycles per run and three independent replicates under each condition. While this protocol is in line with established CpHMD methodologies,^40,41^ the overall trajectory lengths may restrict the observation of slower conformational transitions. Second, only the inactive state of β_2_AR was examined in the absence of ligand or G-protein partners; thus, the relevance of the observed protonation-dependent effects to active-state signaling remains to be established. Future work extending simulations to longer time scales and different receptor states will be essential to validate and expand these observations to to better understand broader allosteric communication within β_2_AR. Incorporating extended microsecond-scale simulations or enhanced-sampling techniques could enable the application of advanced network-based methods, such as shortest-path communication maps,^82^ dynamic correlation networks,^83^ or cryptic-pocket analysis^84^ and allostery-focused disease studies that underscore how mutations or altered signaling partners modulate these networks,^85^ to further interrogate global allosteric pathways. These complementary approaches would provide an expanded view of how protonation-driven local perturbations propagate across the receptor’s energy landscape. Integrating such methodologies in follow-up studies may therefore deepen our understanding of pH-dependent GPCR regulation beyond the nanosecond regime explored here.

## Supplementary Material



## Data Availability

Protein trajectory PDB files for each repeated run and each condition, consisting of 2500 frames (0.04 ns/frame), along with the Python, C, and TCL scripts used, are publicly available on Zenodo at DOI: 10.5281/zenodo.16784477. Python MDAnalysis^86^ and MDTraj^87^ libraries were used for trajectory analysis, and the PyMOL Molecular Graphics System, Version 3.1.1,^88^ Schrödinger, LLC, was used to generate protein structure representations.

## Notes

PropKa v3.0 pKa values, initial CpHMD reference states, protonation–deprotonation fractions, two-way ANOVA results, and minimum Na+ distance analyses (Tables S1–S5); running averages of λ-coordinates, RMSF-based trajectory stability assessment, principal component analysis, all-residue and loop-specific (ECL2 and ICL3) hydrogen bonding patterns, sequence-separation distributions at multiple MI thresholds, dihedral MI heatmaps, and residue-wise ΔMI comparisons (Figures S1–S12) (PDF)

## References

[1] Alberts, B. ; Heald, R. ; Johnson, A. ; Morgan, D. ; Raff, M. ; Roberts, K. ; Walter, P. Molecular Biology of the Cell: Seventh International Student Ed. with Registration Card; WW Norton & Company, 2022.

[2] Casey J. R., Grinstein S., Orlowski J. (2010). Sensors and regulators of intracellular
pH. Nat. Rev. Mol. Cell Biol..

[3] Beasley D. E., Koltz A. M., Lambert J. E., Fierer N., Dunn R. R. (2015). The evolution
of stomach acidity and its relevance to the human microbiome. PLoS One.

[4] Fallingborg J. (1999). Intraluminal
pH of the human gastrointestinal tract. Danish
Med. Bull..

[5] Boelsma E., Van de Vijver L. P., Goldbohm R. A., Klöpping-Ketelaars I. A., Hendriks H. F., Roza L. (2003). Human skin condition and its associations
with nutrient concentrations in serum and diet. Am. J. Clin. Nutr..

[6] Effros R. M., Chinard F. P. (1969). The in vivo pH of the extravascular space of the lung. J. Clin. Invest..

[7] Bruno, C. M. ; Valenti, M. Acid-base disorders in patients with chronic obstructive pulmonary disease: a pathophysiological review BioMed. Res. Int. 2012. 10.1155/2012/915150 PMC3303884 22500110

[8] Seifter J. L., Chang H.-Y. (2017). Disorders of acid-base
balance: new perspectives. Kidney Dis..

[9] Aoi, W. ; Marunaka, Y. Importance of pH homeostasis in metabolic health and diseases: crucial role of membrane proton transport. BioMed. Res. Int. 2014, 2014. 10.1155/2014/598986. PMC4180894 25302301

[10] Kellum J. A. (2000). Determinants
of blood pH in health and disease. Crit. Care.

[11] Schwalfenberg G. K. (2012). The alkaline
diet: is there evidence that an alkaline pH diet benefits health. J. Environ. Public Health.

[12] Ghanouni P., Schambye H., Seifert R., Lee T. W., Rasmussen S. G., Gether U., Kobilka B. K. (2000). The effect
of pH on *β*2 adrenoceptor function: Evidence
for protonation-dependent activation. J. Biol.
Chem..

[13] Palczewski K., Kumasaka T., Hori T., Behnke C. A., Motoshima H., Fox B. A., Trong I. L., Teller D. C., Okada T., Stenkamp R. E. (2000). Crystal structure of rhodopsin: AG protein-coupled
receptor. Science.

[14] Buck L., Axel R. (1991). A novel multigene family
may encode odorant receptors: a molecular
basis for odor recognition. Cell.

[15] Kobilka B. K. (2007). G protein
coupled receptor structure and activation. Biochim.
Biophys. Acta, Biomembr.

[16] Gether U. (2000). Uncovering
molecular mechanisms involved in activation of G protein-coupled receptors. Endocrine Rev..

[17] Murphy P. M. (1996). Chemokine
receptors: structure, function and role in microbial pathogenesis. Cytokine Growth Factor Rev..

[18] Brodde O.-E., Michel M. C. (1999). Adrenergic and muscarinic receptors
in the human heart. Pharmacol. Rev..

[19] Deupi X., Kobilka B. (2007). Activation of G protein–coupled
receptors. Adv. Protein Chem..

[20] Rosenbaum D. M., Rasmussen S. G., Kobilka B. K. (2009). The structure and function of G-protein-coupled
receptors. Nature.

[21] Koch W. J., Lefkowitz R., Rockman H. (2000). Functional consequences of altering
myocardial adrenergic receptor signaling. Annu.
Rev. Physiol..

[22] Kohm A. P., Sanders V. M. (2001). Norepinephrine and *β*2-adrenergic
receptor stimulation regulate CD4+ T and B lymphocyte function in
vitro and in vivo. Pharmacol. Rev..

[23] Liu X., Wu W. K., Yu L., Sung J. J., Srivastava G., Zhang S. T., Cho C. H. (2008). Epinephrine
stimulates esophageal
squamous-cell carcinoma cell proliferation via *β*-adrenoceptor-dependent transactivation of extracellular signal-regulated
kinase/cyclooxygenase-2 pathway. J. Cell. Biochem..

[24] Shang Z. J., Liu K., Liang D. F. (2009). Expression
of *β*2-adrenergic
receptor in oral squamous cell carcinoma. J.
Oral Pathol. Med..

[25] Dror R. O., Arlow D. H., Maragakis P., Mildorf T. J., Pan A. C., Xu H., Borhani D. W., Shaw D. E. (2011). Activation mechanism of the *β* 2-adrenergic receptor. Proc.
Natl. Acad. Sci. U.S.A..

[26] Barreto C. A. V., Vitorino J. N., Reis P. B., Machuqueiro M., Moreira I. S. (2024). p K a Calculations of GPCRs: Understanding Protonation
States in Receptor Activation. J. Chem. Inf.
Model..

[27] Strohman M. J., Maeda S., Hilger D., Masureel M., Du Y., Kobilka B. (2019). Local membrane charge regulates *β*2 adrenergic receptor coupling to Gi3. Nat.
Commun..

[28] Kostikas K., Papatheodorou G., Ganas K., Psathakis K., Panagou P., Loukides S. (2002). pH in expired
breath condensate of
patients with inflammatory airway diseases. Am. J. Respir. Crit. Care Med..

[29] Koczulla A.-R., Noeske S., Herr C., Jörres R. A., Römmelt H., Vogelmeier C., Bals R. (2010). Acute
and chronic effects of smoking on inflammation markers in exhaled
breath condensate in current smokers. Respiration.

[30] Mountain R. D., Heffner J. E., Brackett N. C., Sahn S. A. (1990). Acid-base
disturbances in acute asthma. Chest.

[31] Stather D. R., Stewart T. E. (2005). Clinical review:
Mechanical ventilation in severe asthma. Crit.
Care.

[32] Tang X. X., Ostedgaard L. S., Hoegger M. J., Moninger T. O., Karp P. H., McMenimen J. D., Choudhury B., Varki A., Stoltz D. A., Welsh M. J. (2016). Acidic
pH increases airway surface liquid viscosity
in cystic fibrosis. J. Clin. Invest..

[33] Meyer E. F., Swanson S. M., Williams J. A. (2000). Molecular
modelling and drug design. Pharmacol. Ther..

[34] Mortier J., Rakers C., Bermudez M., Murgueitio M. S., Riniker S., Wolber G. (2015). The impact of molecular
dynamics
on drug design: applications for the characterization of ligand–macromolecule
complexes. Drug Discovery Today.

[35] Jones, T. ; Kjeldgaard, M. Methods in Enzymology; Elsevier, 1997; Vol. 277, pp 173–208. 18488310 10.1016/s0076-6879(97)77012-5

[36] Barcellos G. B., Pauli I., Caceres R. A., Macedo Timmers L. F. S., Dias R., de Azevedo W. F. (2008). Molecular modeling
as a tool for drug discovery. Curr. Drug Targets.

[37] De
Vivo M., Masetti M., Bottegoni G., Cavalli A. (2016). Role of molecular dynamics
and related methods in drug discovery. J. Med.
Chem..

[38] Liu X., Shi D., Zhou S., Liu H., Liu H., Yao X. (2018). Molecular
dynamics simulations and novel drug discovery. Expert Opin. Drug Discovery.

[39] Zhao H., Caflisch A. (2015). Molecular dynamics in drug design. Eur. J. Med. Chem..

[40] Radak B. K., Chipot C., Suh D., Jo S., Jiang W., Phillips J. C., Schulten K., Roux B. (2017). Constant-pH
molecular
dynamics simulations for large biomolecular systems. J. Chem. Theory Comput..

[41] Radak, B. K. ; Roux, B. Efficiency in nonequilibrium molecular dynamics Monte Carlo simulations. J. Chem. Phys. 2016, 145. 10.1063/1.4964288. PMC5055532 27782441

[42] Martins
de Oliveira V., Liu R., Shen J. (2022). Constant pH molecular
dynamics simulations: Current status and recent applications. Curr. Opin. Struct. Biol..

[43] Pieri E., Ledentu V., Sahlin M., Dehez F., Olivucci M., Ferré N. (2019). CpHMD-Then-QM/MM identification of
the amino acids
responsible for the anabaena sensory rhodopsin pH-dependent electronic
absorption spectrum. J. Chem. Theory Comput..

[44] Vo Q. N., Mahinthichaichan P., Shen J., Ellis C. R. (2021). How *μ*-opioid
receptor recognizes fentanyl. Nat.
Commun..

[45] Li C., Yue Z., Newstead S., Voth G. A. (2022). Proton coupling and the multiscale
kinetic mechanism of a peptide transporter. Biophys. J..

[46] Jansen, A. ; Bauer, P. ; Howard, R. J. ; Hess, B. ; Lindahl, E. Constant-pH Molecular Dynamics Simulations of Closed and Open States of a Proton-gated Ion Channel bioRxiv 2023, p 2023 11. 10.1016/j.bpj.2026.08.019 42638311

[47] Moe S., Radak B. K., Chipot C., Roux B. (2025). Simulating biomolecules
with the variable protonation state: A tutorial for constant-pH molecular
dynamics in NAMD. J. Phys. Chem. B.

[48] Cherezov V., Rosenbaum D. M., Hanson M. A., Rasmussen S. G., Thian F. S., Kobilka T. S., Choi H.-J., Kuhn P., Weis W. I., Kobilka B. K., Stevens R. C. (2007). High-resolution
crystal structure of an engineered human *β*2-adrenergic
G protein–coupled receptor. Science.

[49] Webb B., Sali A. (2016). Comparative protein
structure modeling using MODELLER. Curr. Protoc.
Bioinf..

[50] Ozcan O., Uyar A., Doruker P., Akten E. D. (2013). Effect of intracellular
loop 3 on intrinsic dynamics of human *β* 2-adrenergic
receptor. BMC Struct. Biol..

[51] Ozgur C., Doruker P., Akten E. D. (2016). Investigation of allosteric coupling
in human *β* 2-adrenergic receptor in the presence
of intracellular loop 3. BMC Struct. Biol..

[52] Humphrey W., Dalke A., Schulten K. (1996). VMD: visual
molecular dynamics. J. Mol. Graphics.

[53] Best R. B., Zhu X., Shim J., Lopes P. E., Mittal J., Feig M., MacKerell A. D. (2012). Optimization of the additive CHARMM
all-atom protein force field targeting improved sampling of the backbone *ϕ*, *ψ* and side-chain *χ*1 and *χ*2 dihedral angles. J. Chem. Theory Comput..

[54] Kučerka N., Nieh M.-P., Katsaras J. (2011). Fluid phase lipid areas and bilayer
thicknesses of commonly used phosphatidylcholines as a function of
temperature. Biochim. Biophys. Acta, Biomembr..

[55] Celebi M., Akten E. D. (2022). Altered dynamics
of S. aureus phosphofructokinase via
bond restraints at two distinct allosteric binding sites. J. Mol. Biol..

[56] Sogunmez N., Akten E. D. (2020). Distinctive communication networks
in inactive states
of *β*2-adrenergic receptor: mutual information
and entropy transfer analysis. Proteins:Struct.,
Funct., Bioinf..

[57] Sogunmez N., Akten E. D. (2022). Information Transfer in Active States of Human *β*2-Adrenergic Receptor via Inter-Rotameric Motions
of Loop Regions. Appl. Sci..

[58] Ponder J. W., Richards F. M. (1987). Tertiary templates
for proteins: use of packing criteria
in the enumeration of allowed sequences for different structural classes. J. Mol. Biol..

[59] McClendon C. L., Friedland G., Mobley D. L., Amirkhani H., Jacobson M. P. (2009). Quantifying correlations between allosteric sites in
thermodynamic ensembles. J. Chem. Theory Comput..

[60] DuBay K. H., Bothma J. P., Geissler P. L. (2011). Long-range
intra-protein communication
can be transmitted by correlated side-chain fluctuations alone. PLoS Comput. Biol..

[61] Grassberger P. (1988). Finite sample
corrections to entropy and dimension estimates. Phys. Lett. A.

[62] Herzel H., Schmitt A. O., Ebeling W. (1994). Finite sample effects
in sequence
analysis. Chaos, Solitons Fractals.

[63] Rasmussen S. G. F., DeVree B. T., Zou Y., Kruse A. C., Chung K. Y., Kobilka T. S., Thian F. S., Chae P. S., Pardon E., Calinski D. (2011). Crystal
structure of the *β*2 adrenergic receptor–Gs
protein complex. Nature.

[64] Weis W. I., Kobilka B. K. (2018). The molecular basis
of G protein–coupled receptor
activation. Annu. Rev. Biochem..

[65] Ballesteros, J. A. ; Weinstein, H. Methods in Neuroscience; Elsevier, 1995; Vol. 25, pp 366–428.

[66] Herrera L. P. T., Andreassen S. N., Caroli J., Rodríguez-Espigares I., Kermani A. A., Keserü G. M., Kooistra A. J., Pándy-Szekeres G., Gloriam D. E. (2025). GPCRdb in 2025: adding odorant receptors, data mapper,
structure similarity search and models of physiological ligand complexes. Nucleic Acids Res..

[67] Wheatley M., Wootten D., Conner M. T., Simms J., Kendrick R., Logan R. T., Poyner D. R., Barwell J. (2012). Lifting the lid on
GPCRs: the role of extracellular loops. Br.
J. Pharmacol..

[68] Peeters M., Van Westen G., Li Q., IJzerman A. (2011). Importance of the extracellular
loops in G protein-coupled receptors for ligand recognition and receptor
activation. Trends Pharmacol. Sci..

[69] Askalan R., Richardson P. J. (1994). Role of
histidine residues in the adenosine A2a receptor
ligand binding site. J. Neurochem..

[70] Ballabio F., Capelli R. (2025). Constant-pH simulation
of the human *β*2 adrenergic receptor inactivation. J. Chem.
Inf. Model..

[71] Sadler F., Ma N., Ritt M., Sharma Y., Vaidehi N., Sivaramakrishnan S. (2023). Autoregulation
of GPCR signalling through the third intracellular loop. Nature.

[72] Woolley M. J., Conner A. C. (2017). Understanding the common themes and
diverse roles of
the second extracellular loop (ECL2) of the GPCR super-family. Mol. Cell. Endocrinol..

[73] Rasmussen S. G. F., Choi H.-J., Fung J. J., Pardon E., Casarosa P., Chae P. S., DeVree B. T., Rosenbaum D. M., Thian F. S., Kobilka T. S. (2011). Structure of a nanobody-stabilized
active state of the *β*2 adrenoceptor. Nature.

[74] Wess J. (2023). The third
intracellular loop of GPCRs: size matters. Trends
Pharmacol. Sci..

[75] Miao Y., McCammon J. A. (2018). Mechanism of the G-protein mimetic
nanobody binding
to a muscarinic G-protein-coupled receptor. Proc. Natl. Acad. Sci. U.S.A..

[76] Englander S. W., Mayne L., Krishna M. M. (2007). Protein folding and misfolding: mechanism
and principles. Q. Rev. Biophys..

[77] Best R. B., Hummer G., Eaton W. A. (2013). Native
contacts determine protein
folding mechanisms in atomistic simulations. Proc. Natl. Acad. Sci. U.S.A..

[78] Salamanca
Viloria J., Allega M. F., Lambrughi M., Papaleo E. (2017). An optimal distance cutoff for contact-based Protein
Structure Networks using side-chain centers of mass. Sci. Rep..

[79] Noivirt-Brik O., Hazan G., Unger R., Ofran Y. (2013). Non-local residue–residue
contacts in proteins are more conserved than local ones. Bioinformatics.

[80] Mahalingam M., Martínez-Mayorga K., Brown M. F., Vogel R. (2008). Two protonation
switches control rhodopsin activation in membranes. Proc. Natl. Acad. Sci. U.S.A..

[81] Gaudreault F., Chartier M., Najmanovich R. (2012). Side-chain
rotamer changes upon ligand
binding: common, crucial, correlate with entropy and rearrange hydrogen
bonding. Bioinformatics.

[82] Cortada E. P., Osuna S. (2025). Shortest Path Map correlation-based
tool for capturing functionally
relevant allosteric networks and its application in enzyme design. C. R. Chim..

[83] Morra G., Genoni A., Colombo G. (2014). Mechanisms of differential allosteric
modulation in homologous proteins: Insights from the analysis of internal
dynamics and energetics of PDZ domains. J. Chem.
Theory Comput..

[84] Bemelmans M. P., Cournia Z., Damm-Ganamet K. L., Gervasio F. L., Pande V. (2025). Computational
advances in discovering cryptic pockets for drug discovery. Curr. Opin. Struct. Biol..

[85] Berezovsky I. N., Nussinov R. (2025). Allostery in disease:
from mutations, mechanisms, and
signalling partners to diagnostic and drug therapies. J. Mol. Biol..

[86] Gowers, R. J. ; Linke, M. ; Barnoud, J. ; Reddy, T. J. E. ; Melo, M. N. ; Seyler, S. L. ; Domanski, J. ; Dotson, D. L. ; Buchoux, S. ; Kenney, I. M. MDAnalysis: a Python package for the rapid analysis of molecular dynamics simulations. 2019.

[87] Mcgibbon, R. ; Beauchamp, K. ; Schwantes, C. ; Wang, L. ; Hern, C. ; Herrigan, M. ; Lane, T. ; Swails, J. ; Pande, V. MDTraj: a modern, open library for the analysis of molecular dynamics trajectories MDTraj: a modern, open library for the analysis of molecular dynamics trajectories Biorxiv. Org. 2014; Vol. 109 2.

[88] Schrödinger, L. The PyMOL Molecular Graphics System, Version 3.1.1. 2015; Schrödinger, LLC.

