# Supplementary material for: pH-Driven β2AR Dynamics Reveal Loop-Mediated Allosteric Communication
Source: ACS Omega. 2026 Feb 12;11(7):12509–27. doi: 10.1021/acsomega.5c12434 (PMC12947221; doi:10.1021/acsomega.5c12434)
Supplement: Supplementary file 1 [file ao5c12434_si_001.pdf]

Supplementary Material for pH-Driven β_2AR Dynamics Reveal Loop-Mediated Allosteric Communication

Nuray Sogunmez Erdogan*,† and E. Demet Akten‡

†*Kadir Has University, Faculty of Engineering and Natural Sciences, Molecular Biology and Genetics, Istanbul, 34083, Türkiye*

‡*Kadir Has University, Faculty of Engineering and Natural Sciences, Bioinformatics and Genetics, Istanbul, 34083, Türkiye*

E-mail: nuray.erdogan@khas.edu.tr

List of Figures

1 **Running averages of λ-coordinates for each replicate run based on residues with protonation state changes. A)**, Replicate 1 for pH:6.5 (left), pH:7.0 (middle), and pH:8.0 (right), **B)**, Replicate 2 for pH:6.5 (left), pH:7.0 (middle), and pH:8.0 (right), **C)**,Replicate 3 for pH:6.5 (left), pH:7.0 (middle), and pH:8.0 (right). Colors represent indivisual residues with protonation state changes. 13

2 **Assessment of protein trajectory stability across simulations using RMSF analysis. A)**, Root-mean-square fluctuation (RMSF) distributions calculated in 25-ns intervals across the 100-ns production trajectories for all atoms of the protein under four simulation conditions. **B)**, RMSF distributions for the core region with the ICL3 loop excluded, using the same 25-ns interval segmentation. The mean RMSF is shown as a black line, and the standard deviation is indicated by black shading. Average RMSF values range between 1.5–2.0 Å for the full protein (A) but drop below 1.0 Å when ICL3 is excluded, indicating that ICL3 contributes most to the overall fluctuation. Color codes represent temporal segments for each replicate: Replicate 1 – 0–25 ns: blue, 26–50 ns: orange, 51–75 ns: green, 76–100 ns: red; Replicate 2 – 0–25 ns: magenta, 26–50 ns: brown, 51–75 ns: pink, 76–100 ns: gray; Replicate 3 – 0–25 ns: olive green, 26–50 ns: cyan, 51–75 ns: navy blue, 76–100 ns: orange. 14

3 **Principal component analysis (PCA) of molecular dynamics simulations across different conditions. A)** Porcupine plot illustrating dominant motions along the first principal component (PC1), highlighting large-scale conformational changes. **B)** Cumulative variance explained by the first five principal components, showing the proportion of global motion captured. **C)** Root mean square inner product (RMSIP) values comparing the top eigenvectors between trajectories, quantifying similarity in dominant motions across conditions. 15

4 **Analysis of all-residue hydrogen bonding patterns across simulation conditions. Top**, Total count of hydrogen bond persistence (%), showing the fraction of simulation time each residue participates in hydrogen bonding interactions under different pH conditions. **Bottom**, Total number of unique hydrogen bonds identified across trajectories for each condition, reflecting overall interaction diversity. 16

5 **Analysis of hydrogen bonding patterns across simulation conditions on ECL2. Top**, Total count of hydrogen bond persistence (%), showing the fraction of simulation time each ECL2 residue participates in hydrogen bonding interactions under different pH conditions. **Bottom**, Total number of unique hydrogen bonds identified in ECL2 across trajectories for each condition, reflecting overall interaction diversity. 17

6 **Analysis of hydrogen bonding patterns across simulation conditions on ICL3. Top**, Total count of hydrogen bond persistence (%), showing the fraction of simulation time each ICL3 residue participates in hydrogen bonding interactions under different pH conditions. **Bottom**, Total number of unique hydrogen bonds identified in ICL3 across trajectories for each condition, reflecting overall interaction diversity. 18

7 **Sequence-separation distribution of residue pairs with MI ≥0.15.** Histogram showing the distribution of sequence separations ($|i - j|$) for all residue pairs MI greater than 0.15. Residue pairs were grouped into bins of width 5 to visualize how strongly coupled positions are distributed along the primary sequence. **(Top left)**, Control, **(Top right)**, pH:6.5, **(Bottom left)**, pH:7.0, and **(Bottom right)**, pH:8.0. 19

8 **Sequence-separation distribution of residue pairs with MI ≥0.2.** Histogram showing the distribution of sequence separations ($|i-j|$) for all residue pairs MI greater than 0.2. Residue pairs were grouped into bins of width 5 to visualize how strongly coupled positions are distributed along the primary sequence. **(Top left)**, Control, **(Top right)**, pH:6.5, **(Bottom left)**, pH:7.0, and **(Bottom right)**, pH:8.0. 19

9 **Sequence-separation distribution of residue pairs with MI ≥0.25.** Histogram showing the distribution of sequence separations ($|i - j|$) for all residue pairs MI greater than 0.25. Residue pairs were grouped into bins of width 5 to visualize how strongly coupled positions are distributed along the primary sequence. **(Top left)**, Control, **(Top right)**, pH:6.5, **(Bottom left)**, pH:7.0, and **(Bottom right)**, pH:8.0. 20

10 **Sequence-separation distribution of residue pairs with MI ≥0.3.** Histogram showing the distribution of sequence separations ($|i-j|$) for all residue pairs MI greater than 0.3. Residue pairs were grouped into bins of width 5 to visualize how strongly coupled positions are distributed along the primary sequence. **(Top left)**, Control, **(Top right)**, pH:6.5, **(Bottom left)**, pH:7.0, and **(Bottom right)**, pH:8.0. 20

11 **MI heatmaps for dihedral angles. (Top left)**, Control, **(Top right)**, pH:6.5, **(Bottom left)**, pH:7.0, and **(Bottom right)**, pH:8.0. 21

12 **Difference in average MI per residue between CpHMD simulations and the Control.** Shown are ΔMI values for **(Top),** pH:6.5 – Control, textbf(Middle), pH:7.0 – Control, and **(Bottom),** pH:8.0 – Control. Green indicates no change (ΔMI=0), blue indicates increased MI (ΔMI>0), and red indicates decreased MI (ΔMI<0) compared to Control. 22

List of Tables

1 PropKa v3.0 pKa_i results for the initial snapshot of the previously run inactive β_2AR simulation. 6

2 Initial CpHMD reference states and intrinsic $pK_{a,ref}$ values for titratable residues in the inactive β_2AR. 6

3 Protonation-Deprotonation Fractions of Titratable Residues Across Trajectories and pH Conditions (NC = No Change) 7

4 Two-way ANOVA with Tukey HSD Test 11

5 Minimum Na^+ distance to Asp79$^{2.50}$ and Asp113$^{3.32}$ and occupancy within 5 Å across three replicas for each condition. (mindist = minimum distance, Occ. = Occupancy) 12

Table S1: PropKa v3.0 pKa_i results for the initial snapshot of the previously run inactive β_2AR simulation.

**Res**	**ID**	**pKa$_i$**	**Res**	**ID**	**pKa$_i$**	**Res**	**ID**	**pKa$_i$**	**Res**	**ID**	**pKa$_i$**	**Res**	**ID**	**pKa$_i$**
ASP	79	6.2	GLU	62	4.5	HIS	93	5.49	CYS	77	10.24	LYS	60	10.41
ASP	113	6.55	GLU	107	4.21	HIS	172	6.82	CYS	106	ND	LYS	97	10.42
ASP	130	4.24	GLU	122	5.64	HIS	178	6.26	CYS	116	13.01	LYS	140	11.43
ASP	192	3.3	GLU	180	4.55	HIS	241	6.35	CYS	125	11.16	LYS	147	10.22
ASP	234	3.58	GLU	188	4.69	HIS	256	6.43	CYS	184	ND	LYS	149	10.41
ASP	251	2.96	GLU	225	2.88	HIS	269	5.85	CYS	190	ND	LYS	227	10.53
ASP	300	3.82	GLU	237	3.88	HIS	296	5.75	CYS	191	ND	LYS	232	10.39
ASP	331	5.48	GLU	249	3.86				CYS	265	8.92	LYS	235	11.32
			GLU	268	3.89				CYS	285	10.67	LYS	263	10.68
			GLU	306	3.63				CYS	327	9.66	LYS	267	10.58
			GLU	338	4.63				CYS	341	9.0	LYS	270	10.05
												LYS	273	10.21
												LYS	305	11.3

Table S2: Initial CpHMD reference states and intrinsic $pK_{a,ref}$ values for titratable residues in the inactive β_2AR.

**Res**	**ID**	**State**	**pK$_{a,ref}$**	**Res**	**ID**	**State**	**pK$_{a,ref}$**	**Res**	**ID**	**State**	**pK$_{a,ref}$**	**Res**	**ID**	**State**	**pK$_{a,ref}$**
LYS	60	P	10.4	GLU	62	D	4.4	CYS	77	N	9.5	ASP	79	D	4.0
HIS	93	P	6.5–7.0	LYS	97	P	10.4	CYS	106	ND	ND	GLU	107	D	4.4
ASP	113	D	4.0	CYS	116	N	9.5	GLU	122	D	4.4	CYS	125	N	9.5
ASP	130	D	4.0	LYS	140	P	10.4	LYS	147	P	10.4	LYS	149	P	10.4
HIS	172	P	6.5–7.0	HIS	178	E	6.5–7.0	GLU	180	D	4.4	CYS	184	ND	ND
GLU	188	D	4.4	CYS	190	ND	ND	CYS	191	ND	ND	ASP	192	D	4.0
GLU	225	D	4.4	LYS	227	P	10.4	LYS	232	P	10.4	ASP	234	D	4.0
LYS	235	P	10.4	GLU	237	D	4.4	HIS	241	P	6.5–7.0	GLU	249	D	4.4
ASP	251	D	4.0	LYS	263	P	10.4	CYS	265	N	9.5	LYS	267	P	10.4
GLU	268	D	4.4	HIS	269	E	6.5–7.0	LYS	270	P	10.4	LYS	273	P	10.4
CYS	285	N	9.5	HIS	296	E	6.5–7.0	ASP	300	D	4.0	LYS	305	P	10.4
GLU	306	D	4.4	CYS	327	N	9.5	ASP	331	D	4.0	GLU	338	D	4.4
CYS	341	N	9.5												

Table S3: Protonation-Deprotonation Fractions of Titratable Residues Across Trajectories and pH Conditions (NC = No Change)

**Residue**	**pK_a (t=0)**	**Trajectory 1**			**Trajectory 2**			**Trajectory 3**		
		pH 6.5	pH 7.0	pH 8.0	pH 6.5	pH 7.0	pH 8.0	pH 6.5	pH 7.0	pH 8.0
60:LYS	10.4	NC	NC	NC	NC	NC	NC	NC	NC	NC
62:GLU	4.4	0	0	0	0.39	0.171	0	0.383	0	0
77:CYS	9.5	NC	NC	NC	NC	NC	NC	NC	NC	NC
79:ASP	4.0	NC	NC	NC	NC	NC	NC	NC	NC	NC
93:HIS	6.5	0.971	0.856	0	0.928	0.816	0	0.99	0.851	0.91
97:LYS	10.4	NC	NC	NC	NC	NC	NC	NC	NC	NC
106:CYS	9.5	NC	NC	NC	NC	NC	NC	NC	NC	NC
107:GLU	4.4	NC	NC	NC	NC	NC	NC	NC	NC	NC
113:ASP	4.0	NC	NC	NC	NC	NC	NC	NC	NC	NC
116:CYS	9.5	NC	NC	NC	NC	NC	NC	NC	NC	NC
122:GLU	4.4	0	0	0	0	0	0	0	0.009	0

Continued on next page

Residue	pK$_a$ (t=0)	Trajectory 1			Trajectory 2			Trajectory 3		
		pH 6.5	pH 7.0	pH 8.0	pH 6.5	pH 7.0	pH 8.0	pH 6.5	pH 7.0	pH 8.0
125:CYS	9.5	NC	NC	NC	NC	NC	NC	NC	NC	NC
130:ASP	4.0	NC	NC	NC	NC	NC	NC	NC	NC	NC
140:LYS	10.4	NC	NC	NC	NC	NC	NC	NC	NC	NC
147:LYS	10.4	NC	NC	NC	NC	NC	NC	NC	NC	NC
149:LYS	10.4	NC	NC	NC	NC	NC	NC	NC	NC	NC
172:HIS	6.5	1	1	0	1	0.744	0.731	0.566	0.821	0
178:HIS	6.5	0.768	0.069	0.01	0.055	0.19	0.001	0.875	0.146	0
180:GLU	4.4	NC	NC	NC	NC	NC	NC	NC	NC	NC
184:CYS	9.5	NC	NC	NC	NC	NC	NC	NC	NC	NC
188:GLU	4.4	NC	NC	NC	NC	NC	NC	NC	NC	NC
190:CYS	9.5	1	1	1	1	1	1	1	1	0.998
191:CYS	9.5	NC	NC	NC	NC	NC	NC	NC	NC	NC
192:ASP	4.0	0.043	0	0	0	0	0	0	0	0

Continued on next page

Residue	pK_a (t=0)	Trajectory 1			Trajectory 2			Trajectory 3		
		pH 6.5	pH 7.0	pH 8.0	pH 6.5	pH 7.0	pH 8.0	pH 6.5	pH 7.0	pH 8.0
225:GLU	4.4	NC	NC	NC	NC	NC	NC	NC	NC	NC
227:LYS	10.4	NC	NC	NC	NC	NC	NC	NC	NC	NC
232:LYS	10.4	NC	NC	NC	NC	NC	NC	NC	NC	NC
234:ASP	4.0	NC	NC	NC	NC	NC	NC	NC	NC	NC
235:LYS	10.4	NC	NC	NC	NC	NC	NC	NC	NC	NC
237:GLU	4.4	NC	NC	NC	NC	NC	NC	NC	NC	NC
241:HIS	6.5	0.97	0.755	0.019	1	0.952	0	0.932	0.963	0
249:GLU	4.4	0.998	0.937	0.002	0	0	0	0	0.267	0
251:ASP	4.0	NC	NC	NC	NC	NC	NC	NC	NC	NC
256:HIS	6.5	0.963	0.974	0	1	0.804	0.823	1	0.863	0.883
263:LYS	10.4	NC	NC	NC	NC	NC	NC	NC	NC	NC
265:CYS	9.5	NC	NC	NC	NC	NC	NC	NC	NC	NC
267:LYS	10.4	NC	NC	NC	NC	NC	NC	NC	NC	NC

Continued on next page

**Residue**	**pK_a (t=0)**	**Trajectory 1**			**Trajectory 2**			**Trajectory 3**		
		pH 6.5	pH 7.0	pH 8.0	pH 6.5	pH 7.0	pH 8.0	pH 6.5	pH 7.0	pH 8.0
268:GLU	4.4	0.378	0	0	0	0	0	0.459	0	0
269:HIS	6.5	0.004	0	0	0.048	0.03	0	0.105	0.003	0
270:LYS	10.4	NC	NC	NC	NC	NC	NC	NC	NC	NC
273:LYS	10.4	NC	NC	NC	NC	NC	NC	NC	NC	NC
285:CYS	9.5	NC	NC	NC	NC	NC	NC	NC	NC	NC
296:HIS	6.5	1	1	0	0.818	0.001	0	1	0	0
300:ASP	4.0	NC	NC	NC	NC	NC	NC	NC	NC	NC
305:LYS	10.4	1	1	1	1	0	0.986	1	1	1
306:GLU	4.4	NC	NC	NC	NC	NC	NC	NC	NC	NC
327:CYS	9.5	NC	NC	NC	NC	NC	NC	NC	NC	NC
331:ASP	4.0	NC	NC	NC	NC	NC	NC	NC	NC	NC
338:GLU	4.4	0.834	0	0	0.376	0	0	0.972	0	0
341:CYS	9.5	NC	NC	NC	NC	NC	NC	NC	NC	NC

Table S4: Two-way ANOVA with Tukey HSD Test

group1	group2	meandiff	p-adj	lower	upper	reject
Control	pH 6.5	0.0116	0.1175	-0.0018	0.0249	False
Control	pH 7	-0.0015	0.9909	-0.0149	0.0118	False
Control	pH 8	-0.0031	0.9335	-0.0165	0.0103	False
pH 6.5	pH 7	-0.0131	0.0572	-0.0265	0.0003	False
**pH 6.5**	**pH 8**	**-0.0147**	**0.0252**	**-0.028**	**-0.0013**	**True**
pH 7	pH 8	-0.0016	0.9908	-0.0149	0.0118	False

**ANOVA F-value: 3.2959, p-value: 0.0198*

Table S5: Minimum Na^+ distance to $Asp79^{2.50}$ and $Asp113^{3.32}$ and occupancy within 5 Å across three replicas for each condition. (mindist = minimum distance, Occ. = Occupancy)

**Condition**	**Replica**	$\mathbf{Asp79}^{(2.50)}_{mindist}$ **(Å)**	$\mathbf{Asp79}^{(2.50)}_{Occ.}$ **(%)**	$\mathbf{Asp113}^{(3.32)}_{mindist}$ **(Å)**	$\mathbf{Asp113}^{(3.32)}_{Occ.}$ **(%)**
Control	Trajectory1	10.24	0.0	2.39	99.3
	Trajectory2	11.93	0.0	2.54	98.0
	Trajectory3	26.87	0.0	18.86	5.8
pH:6.5	Trajectory1	11.31	0.0	3.75	93.4
	Trajectory2	10.05	0.0	2.23	100.0
	Trajectory3	20.63	0.0	18.70	0.0
pH:7.0	Trajectory1	10.17	0.0	2.22	100.0
	Trajectory2	11.20	0.0	3.65	89.6
	Trajectory3	19.60	0.0	13.11	41.1
pH:8.0	Trajectory1	9.95	0.0	2.21	100.0
	Trajectory2	11.09	0.0	2.26	100.0
	Trajectory3	15.38	0.0	5.76	78.8

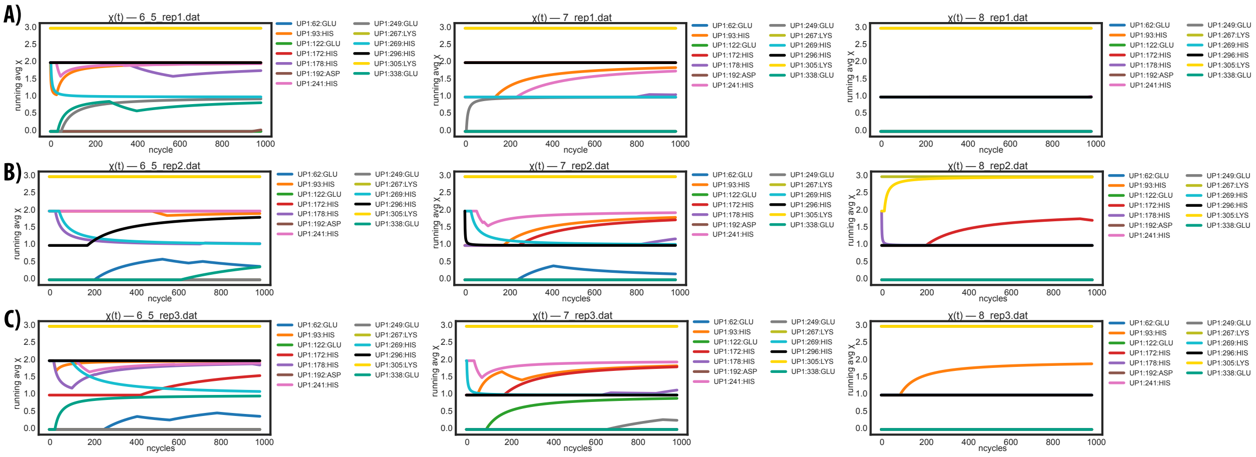

Fig. S1: **Running averages of λ-coordinates for each replicate run based on residues with protonation state changes. A)**, Replicate 1 for pH:6.5 (left), pH:7.0 (middle), and pH:8.0 (right), **B)**, Replicate 2 for pH:6.5 (left), pH:7.0 (middle), and pH:8.0 (right), **C)**,Replicate 3 for pH:6.5 (left), pH:7.0 (middle), and pH:8.0 (right). Colors represent indivisual residues with protonation state changes.

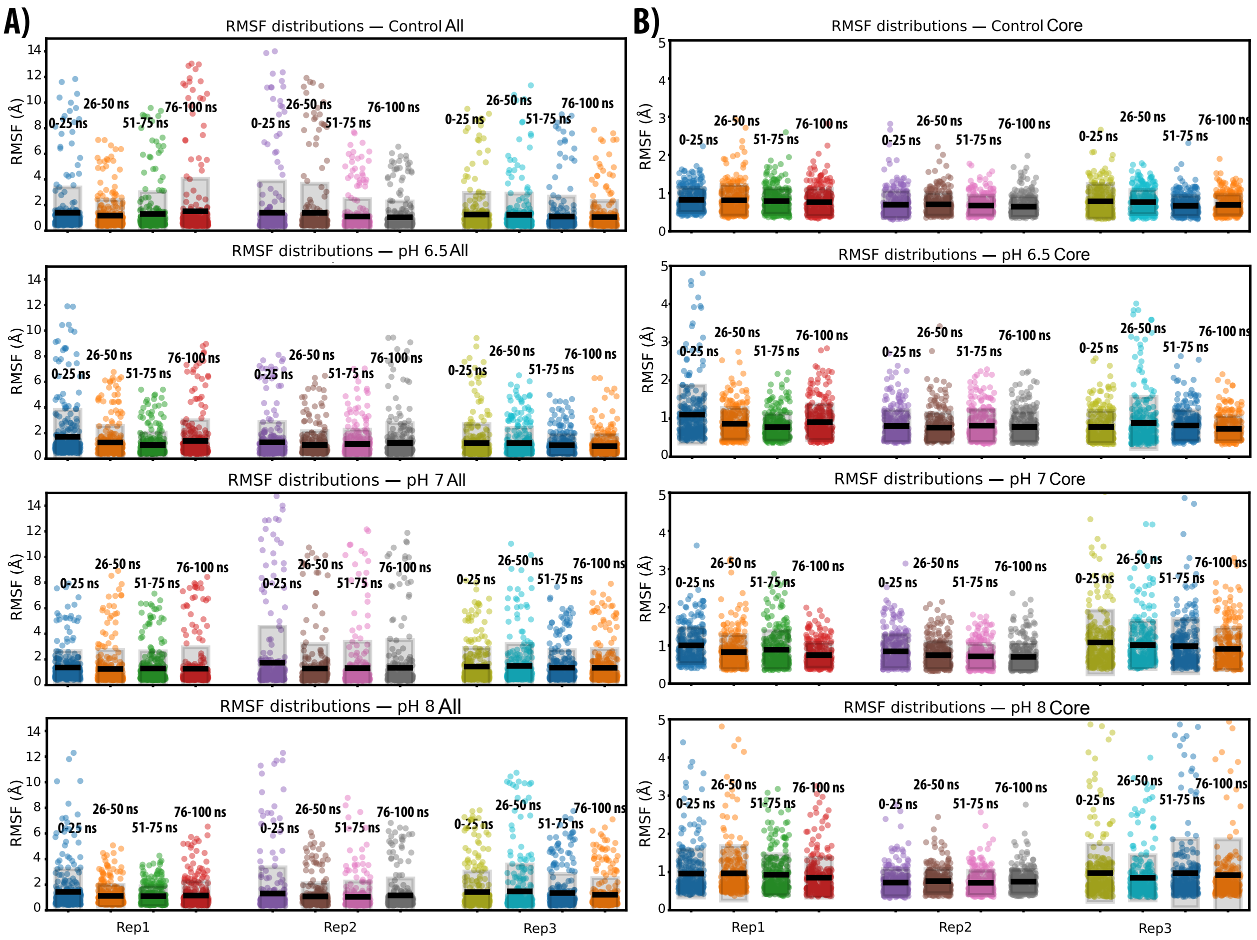

Fig. S2: **Assessment of protein trajectory stability across simulations using RMSF analysis. A)**, Root-mean-square fluctuation (RMSF) distributions calculated in 25-ns intervals across the 100-ns production trajectories for all atoms of the protein under four simulation conditions. **B)**, RMSF distributions for the core region with the ICL3 loop excluded, using the same 25-ns interval segmentation. The mean RMSF is shown as a black line, and the standard deviation is indicated by black shading. Average RMSF values range between 1.5–2.0 Å for the full protein (A) but drop below 1.0 Å when ICL3 is excluded, indicating that ICL3 contributes most to the overall fluctuation. Color codes represent temporal segments for each replicate: Replicate 1 – 0–25 ns: blue, 26–50 ns: orange, 51–75 ns: green, 76–100 ns: red; Replicate 2 – 0–25 ns: magenta, 26–50 ns: brown, 51–75 ns: pink, 76–100 ns: gray; Replicate 3 – 0–25 ns: olive green, 26–50 ns: cyan, 51–75 ns: navy blue, 76–100 ns: orange.

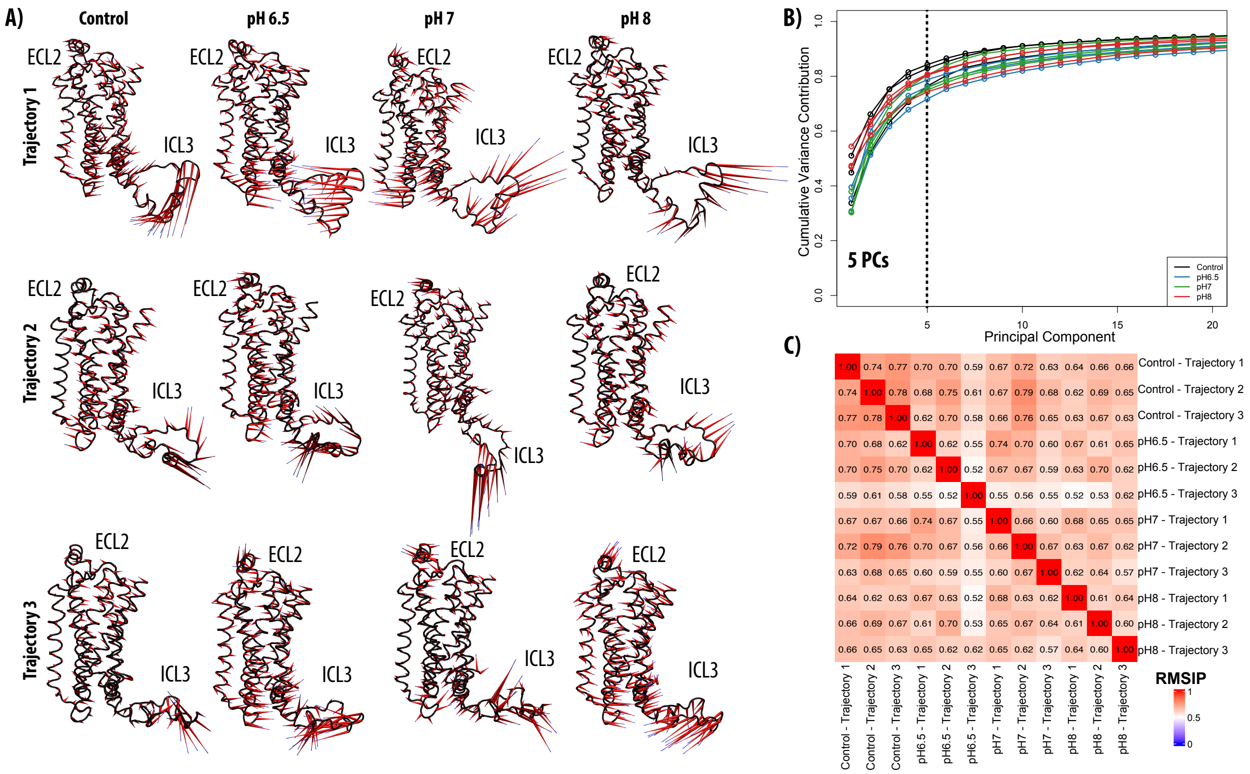

Fig. S3: **Principal component analysis (PCA) of molecular dynamics simulations across different conditions. A)** Porcupine plot illustrating dominant motions along the first principal component (PC1), highlighting large-scale conformational changes. **B)** Cumulative variance explained by the first five principal components, showing the proportion of global motion captured. **C)** Root mean square inner product (RMSIP) values comparing the top eigenvectors between trajectories, quantifying similarity in dominant motions across conditions.

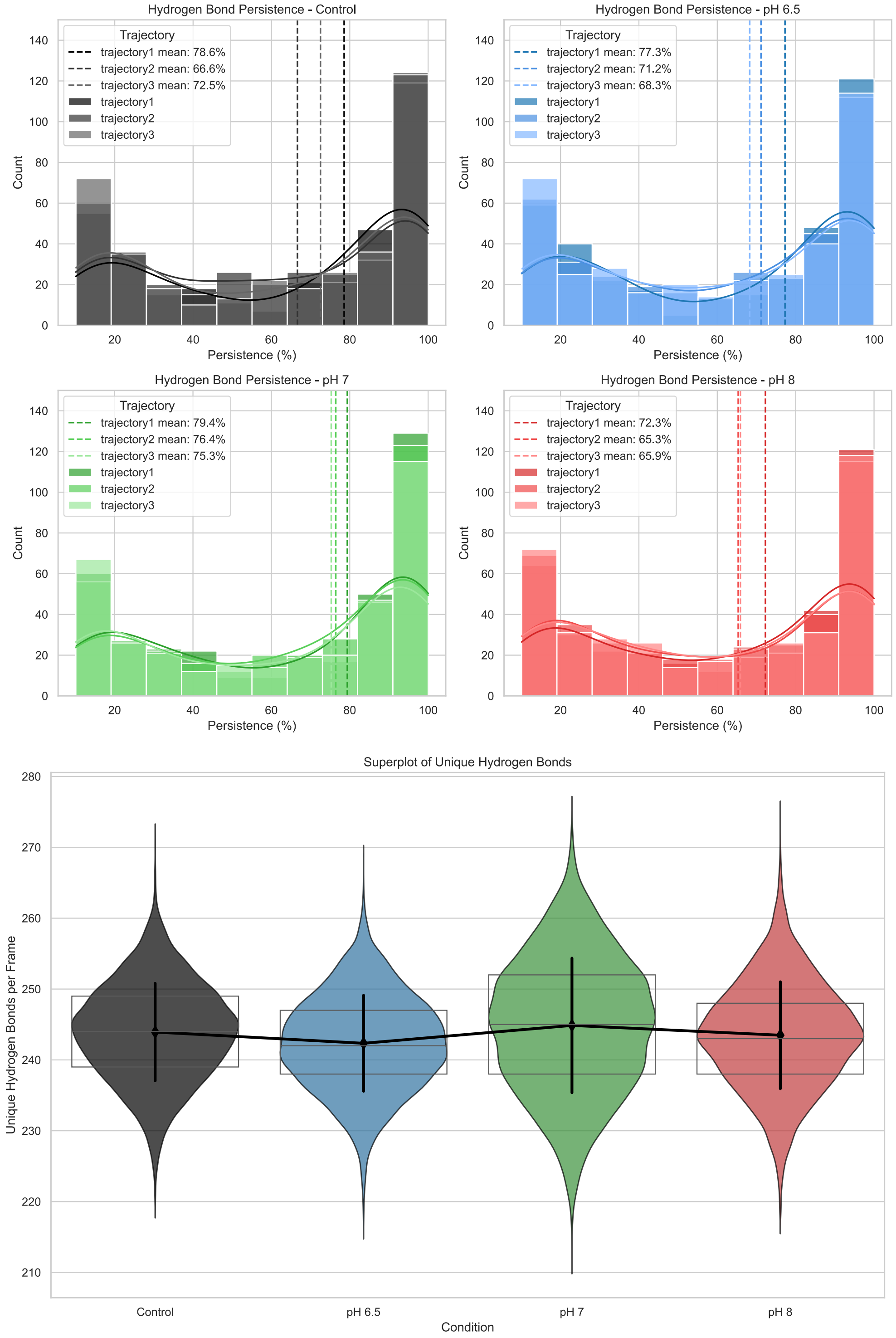

Fig. S4: **Analysis of all-residue hydrogen bonding patterns across simulation conditions.** **Top**, Total count of hydrogen bond persistence (%), showing the fraction of simulation time each residue participates in hydrogen bonding interactions under different pH conditions. **Bottom**, Total number of unique hydrogen bonds identified across trajectories for each condition, reflecting overall interaction diversity.

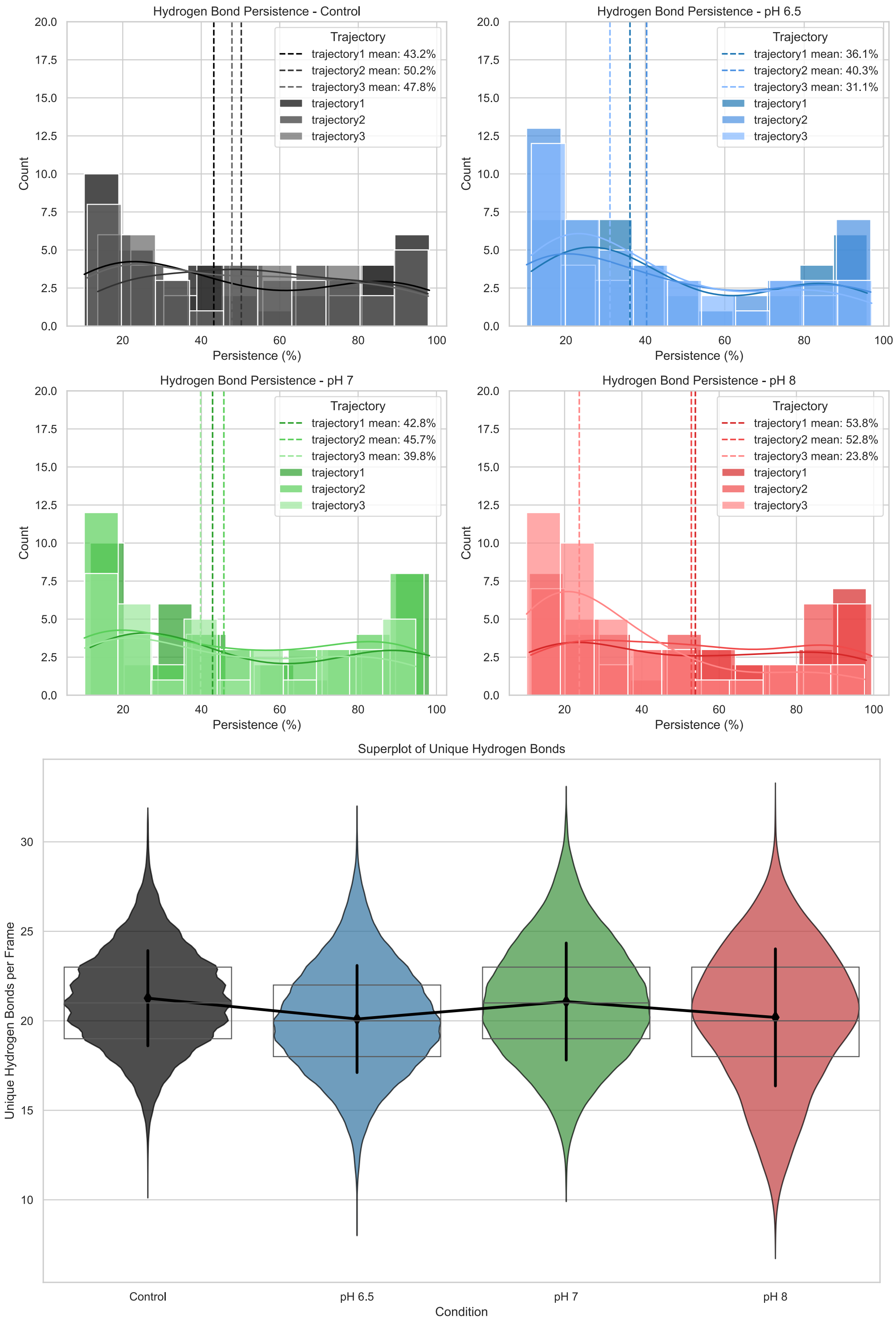

Fig. S5: **Analysis of hydrogen bonding patterns across simulation conditions on ECL2. Top**, Total count of hydrogen bond persistence (%), showing the fraction of simulation time each ECL2 residue participates in hydrogen bonding interactions under different pH conditions. **Bottom**, Total number of unique hydrogen bonds identified in ECL2 across trajectories for each condition, reflecting overall interaction diversity.

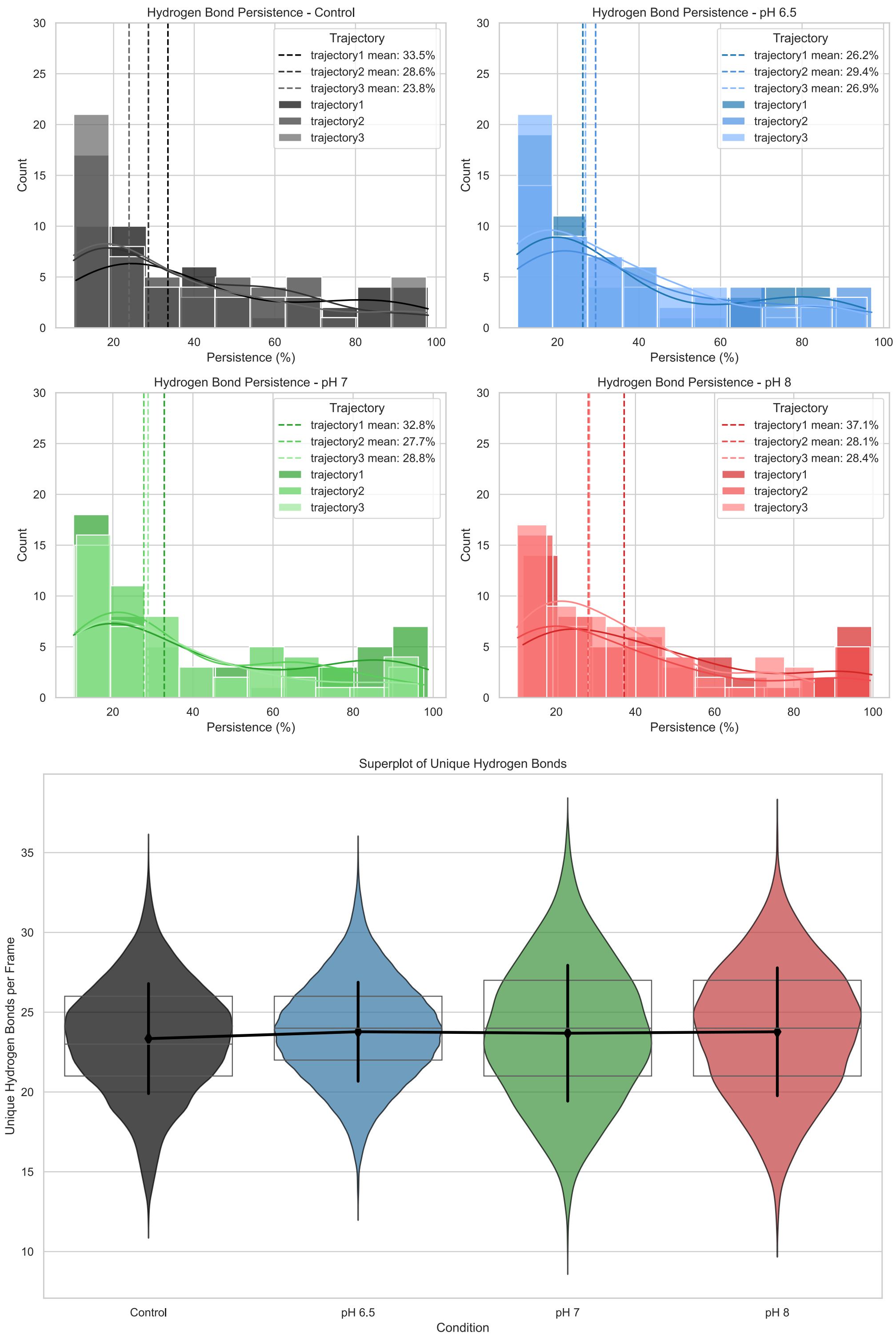

Fig. S6: **Analysis of hydrogen bonding patterns across simulation conditions on ICL3. Top**, Total count of hydrogen bond persistence (%), showing the fraction of simulation time each ICL3 residue participates in hydrogen bonding interactions under different pH conditions. **Bottom**, Total number of unique hydrogen bonds identified in ICL3 across trajectories for each condition, reflecting overall interaction diversity.

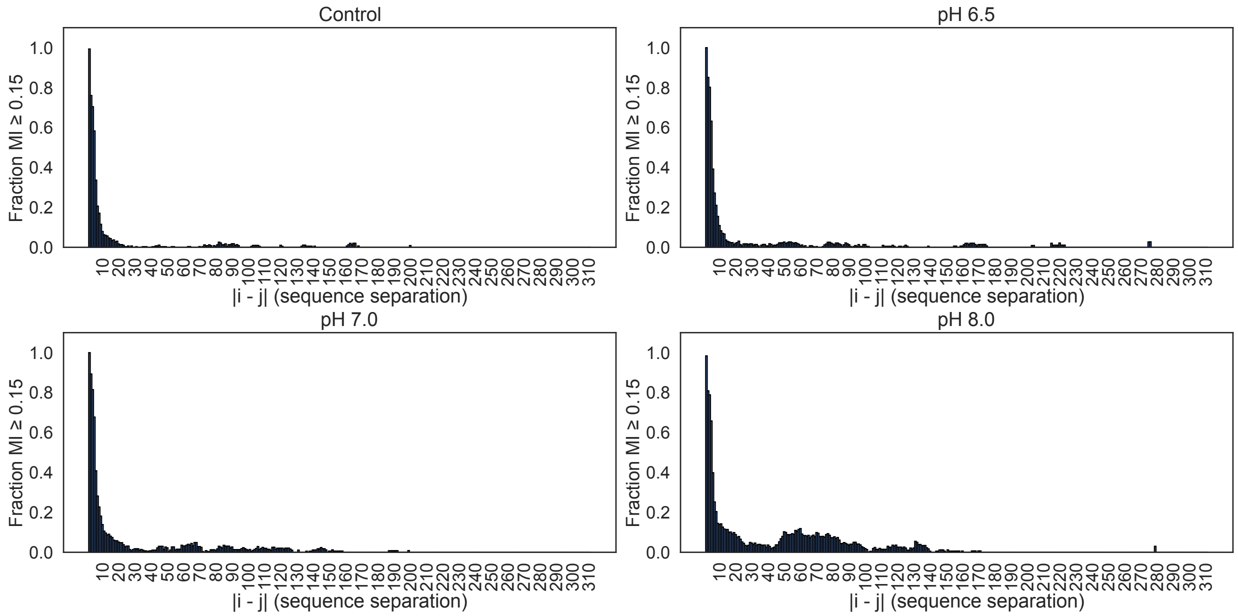

Fig. S7: **Sequence-separation distribution of residue pairs with MI $\geq$0.15.** Histogram showing the distribution of sequence separations ($|i - j|$) for all residue pairs MI greater than 0.15. Residue pairs were grouped into bins of width 5 to visualize how strongly coupled positions are distributed along the primary sequence. **(Top left)**, Control, **(Top right)**, pH:6.5, **(Bottom left)**, pH:7.0, and **(Bottom right)**, pH:8.0.

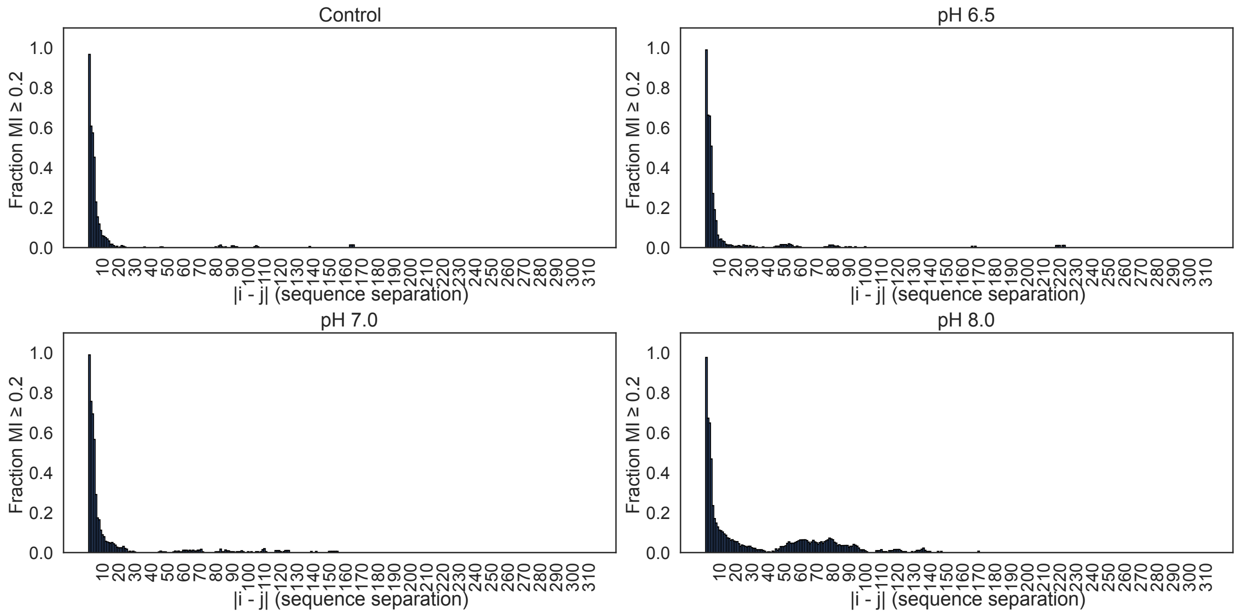

Fig. S8: **Sequence-separation distribution of residue pairs with MI $\geq$0.2.** Histogram showing the distribution of sequence separations ($|i - j|$) for all residue pairs MI greater than 0.2. Residue pairs were grouped into bins of width 5 to visualize how strongly coupled positions are distributed along the primary sequence. **(Top left)**, Control, **(Top right)**, pH:6.5, **(Bottom left)**, pH:7.0, and **(Bottom right)**, pH:8.0.

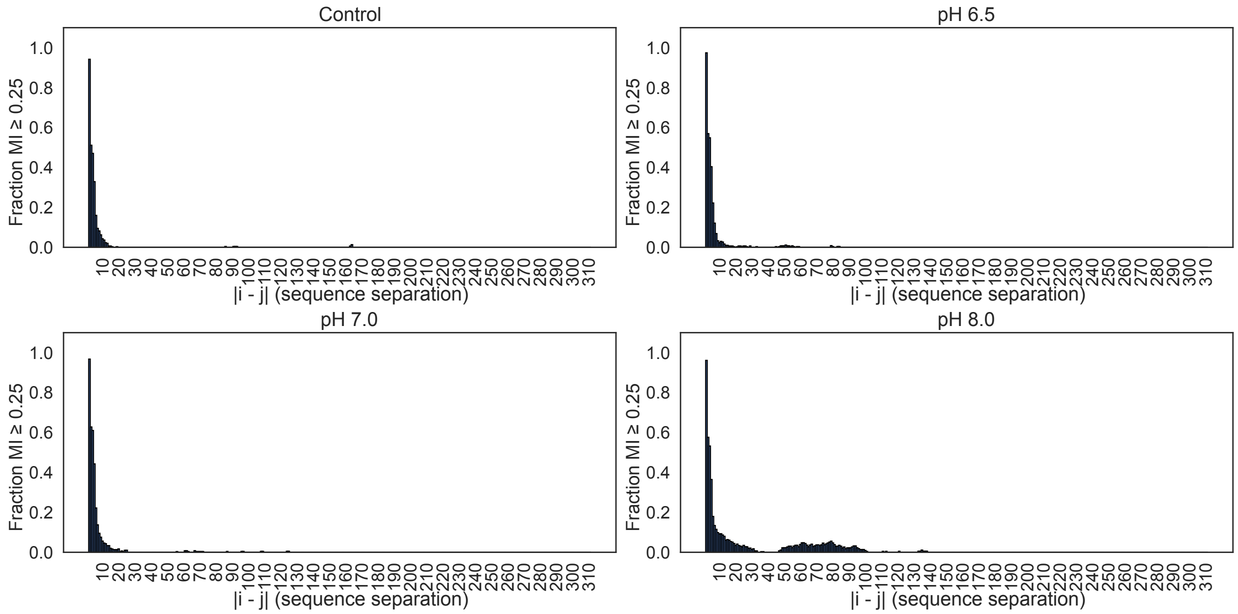

Fig. S9: **Sequence-separation distribution of residue pairs with MI $\geq$0.25.** Histogram showing the distribution of sequence separations ($|i - j|$) for all residue pairs MI greater than 0.25. Residue pairs were grouped into bins of width 5 to visualize how strongly coupled positions are distributed along the primary sequence. **(Top left)**, Control, **(Top right)**, pH:6.5, **(Bottom left)**, pH:7.0, and **(Bottom right)**, pH:8.0.

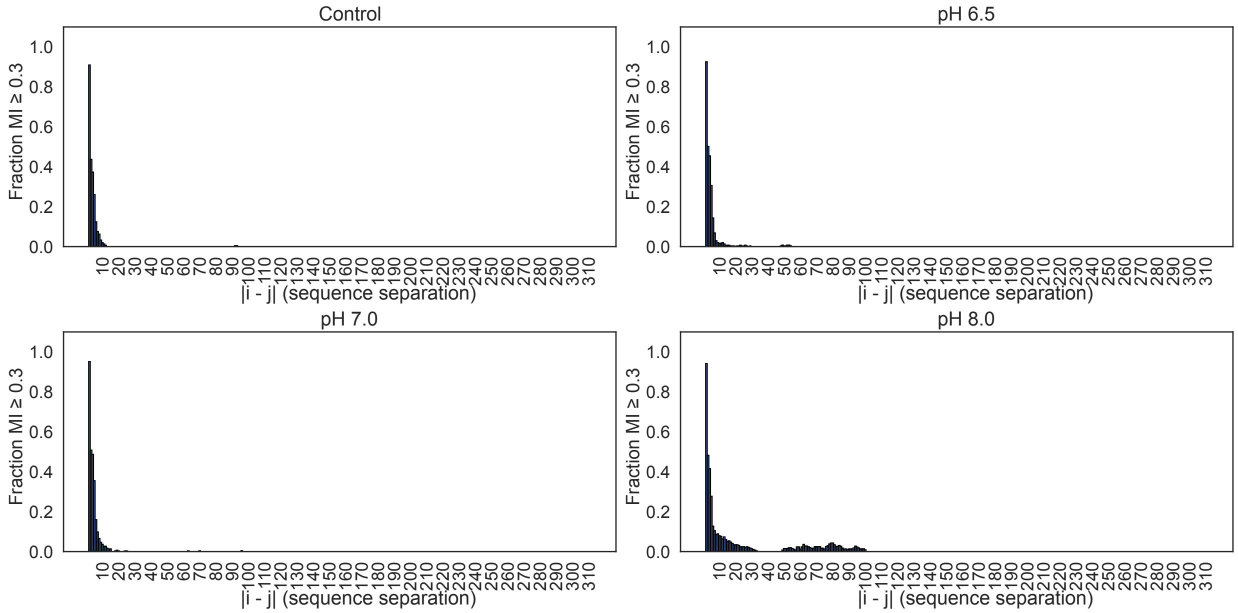

Fig. S10: **Sequence-separation distribution of residue pairs with MI $\geq$0.3.** Histogram showing the distribution of sequence separations ($|i - j|$) for all residue pairs MI greater than 0.3. Residue pairs were grouped into bins of width 5 to visualize how strongly coupled positions are distributed along the primary sequence. **(Top left)**, Control, **(Top right)**, pH:6.5, **(Bottom left)**, pH:7.0, and **(Bottom right)**, pH:8.0.

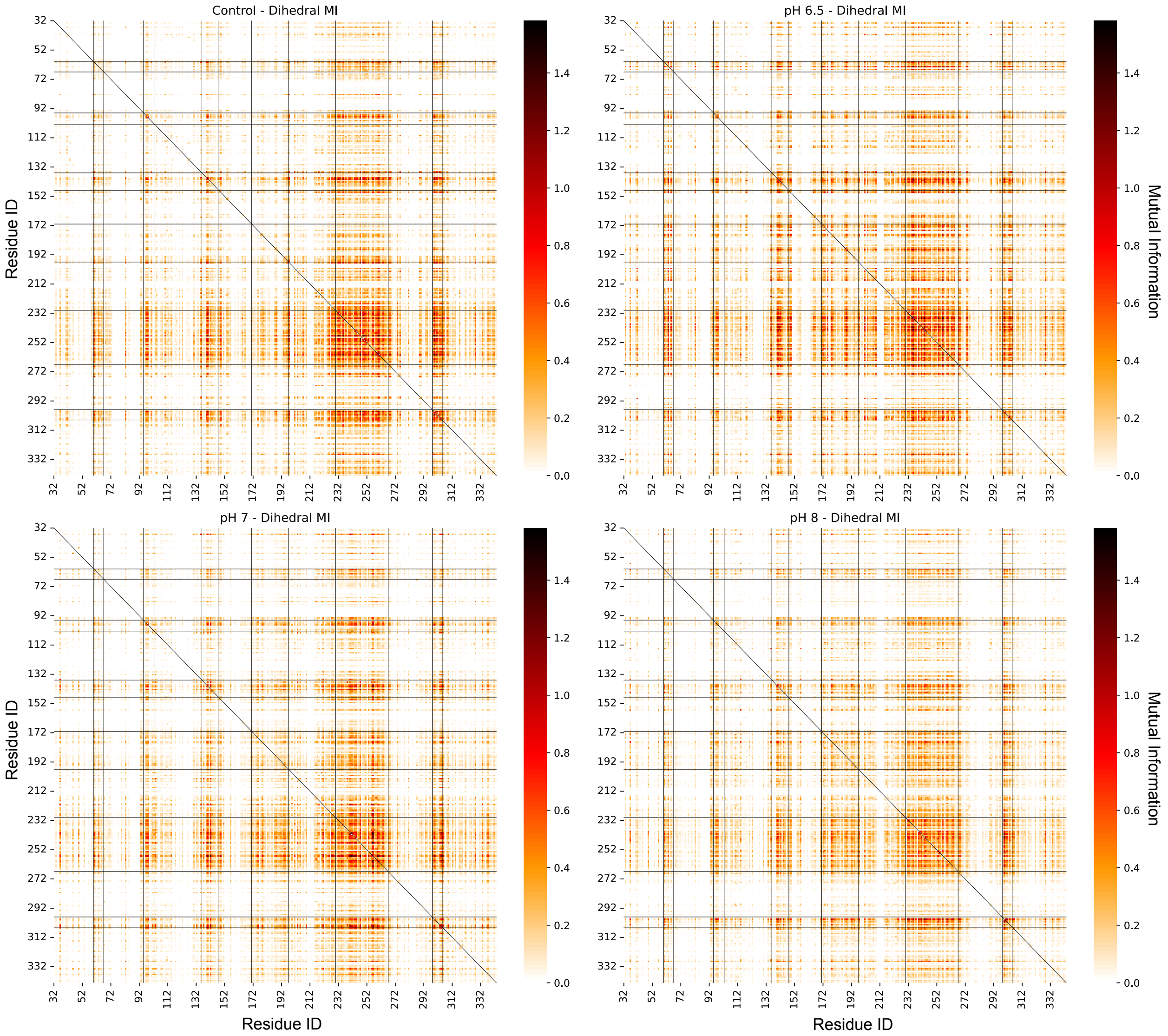

Fig. S11: **MI heatmaps for dihedral angles. (Top left)**, Control, **(Top right)**, pH:6.5, **(Bottom left)**, pH:7.0, and **(Bottom right)**, pH:8.0.

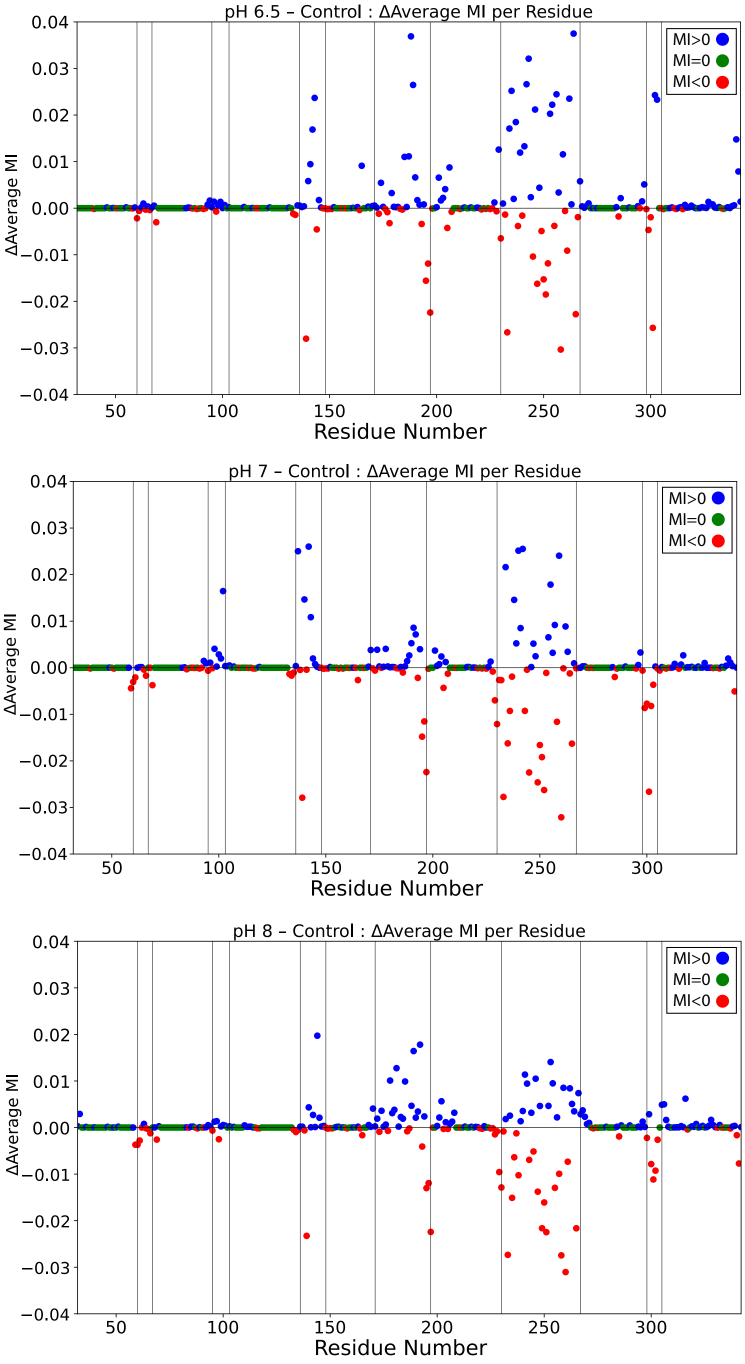

Fig. S12: **Difference in average MI per residue between CpHMD simulations and the Control.** Shown are ΔMI values for **(Top),** pH:6.5 – Control, textbf(Middle), pH:7.0 – Control, and **(Bottom),** pH:8.0 – Control. Green indicates no change (ΔMI=0), blue indicates increased MI (ΔMI>0), and red indicates decreased MI (ΔMI<0) compared to Control.
